# Characterization of the Δ7 Mutant of *Cupriavidus metallidurans* with Deletions of Seven Secondary Metal Uptake Systems

**DOI:** 10.1128/mSystems.00004-16

**Published:** 2016-02-25

**Authors:** Cornelia Große, Martin Herzberg, Marcel Schüttau, Nicole Wiesemann, Gerd Hause, Dietrich H. Nies

**Affiliations:** a Molecular Microbiology, Institute for Biology/Microbiology, Martin-Luther-University Halle-Wittenberg, Halle, Germany; b Microscopy Unit, Biocenter, Martin-Luther-University Halle-Wittenberg, Halle, Germany; University of Rhode Island

**Keywords:** *Cupriavidus*, *Ralstonia*, zinc, cadmium, cobalt

## Abstract

Central to the ability of *Cupriavidus metallidurans* to maintain its metal homoeostasis is the metal transportome, composed of uptake and efflux systems. Seven secondary metal import systems, ZupT, PitA, CorA_1_, CorA_2_, CorA_3_, ZntB, and HoxN, interact and are at the core of the metal uptake transportome. The 7-fold deletion mutant Δ7 (*ΔzupT ΔpitA ΔcorA*_1_ *ΔcorA*_2_ *ΔcorA*_3_ *ΔzntB ΔhoxN*) of parent strain AE104 is still able to maintain its cellular metal content, although at the cost of reduced fitness (M. Herzberg, L. Bauer, A. Kirsten, and D. H. Nies, Metallomics, in press, http://dx.doi.org/10.1039/C5MT00295H). Strain Δ7 does not express genes for backup importers, and so Δ7 should use metal uptake systems also produced in the AE104 parent cells. These systems should be activated in Δ7 by posttranscriptional regulatory processes. The decreased fitness of Δ7 correlated with a zinc-dependent downregulation of the overall metabolic backbone of the cells even at nontoxic external zinc concentrations. Responsible for this decreased fitness of Δ7 was a negative interference of the activity of two P-type ATPases, MgtA and MgtB, which, on the other hand, kept Δ7 at a fitness level higher than that of the Δ9 (Δ7 Δ*mgtA*::*kan ΔmgtB*) mutant strain. This revealed a complicated interplay of the metal uptake transportome of *C. metallidurans*, which is composed of the seven secondary uptake systems, MgtA, MgtB, and yet-unknown components, with cytoplasmic transition metal pools and posttranscriptional regulatory processes.

**IMPORTANCE** Bacteria, including pathogenic strains, need to make use of the metal composition and speciation of their environment to fulfill the requirement of the cytoplasmic metal content and composition. This task is performed by the bacterial metal transportome, composed of uptake and efflux systems. Seven interacting secondary metal uptake systems are at the core of the metal transportome in *C. metallidurans*. This publication verifies that posttranscriptional events are responsible for activation of even more, yet-unknown, metal import systems in the 7-fold deletion mutant Δ7. Two P-type ATPases were identified as new members of the metal uptake transportome. This publication demonstrates the complexity of the metal transportome and the regulatory processes involved.

## Abstract (precis)

Bacteria, including pathogenic strains, need to make use of the metal composition and speciation of their environment to fulfill the requirement of the cytoplasmic metal content and composition. This task is performed by the bacterial metal transportome, composed of uptake and efflux systems. Seven interacting secondary metal uptake systems are at the core of the metal transportome in *C. metallidurans*. This publication verifies that posttranscriptional events are responsible for activation of even more, yet-unknown, metal import systems in the 7-fold deletion mutant Δ7. Two P-type ATPases were identified as new members of the metal uptake transportome. This publication demonstrates the complexity of the metal transportome and the regulatory processes involved.

## INTRODUCTION

*Cupriavidus metallidurans* is able to maintain its metal homoeostasis even in the presence of high external metal concentrations and of mixtures thereof (1–6). Metal efflux systems are central to this capability (3), but also metal uptake systems are essential as their counterparts (2). Together with the efflux systems, the uptake systems constitute the metal transportome, which transforms the environmental metal content, composition, and speciation into the required cytoplasmic mixture of essential metals. Seven secondary transporters form the core of the metal uptake transportome and interact as a battery of redundant importers with low but overlapping substrate specificities (2, 7): (i) the ZIP protein ZupT (TC#1.A.5, transporter classification [8, 9]); (ii) the metal-phosphate importer PitA (TC#2.A.20); (iii) four members of the MIT family, CorA_1_ to CorA_3_ and ZntB (TC#1.A.35); and the NiCoT protein HoxN (TC#2.A.52).

It can be speculated that deletion of the gene for any one of these metal importers will not have any effect because other transporters with overlapping substrate specificities will take over its function. Indeed, deletion of *zupT* in the plasmid-free *C. metallidurans* strain AE104 appears not to compromise net uptake of zinc or other metal cations (2). On closer inspection, however, the Δ*zupT* mutant strain suffers from various defects, indicating that ZupT is required for zinc uptake at low environmental concentrations and for efficient zinc allocation to client proteins (2, 10–12). The number of zinc atoms per cell is lower in the Δ*zupT* mutant cells than in the parent strain AE104, namely, about 20,000 atoms per cell compared to 70,000 atoms in cells cultivated in mineral salts medium without added metals (2, 10). Addition of EDTA, a metal-chelating substance, enhances the metal starvation phenotype of the Δ*zupT* mutant cells and decreases the iron, cobalt, nickel, copper, and zinc content of *C. metallidurans* cells (10). In particular, an operon region encoding a putative zinc chaperone plus paralogs of zinc-dependent proteins is upregulated 115-fold in Δ*zupT* cells cultivated in the presence of 50 µM EDTA but only 24-fold in AE104 cells grown under similar conditions (12). Addition of zinc chloride to the growth medium fills the cellular zinc pool in the parent strain AE104. At 100 µM added Zn(II), a concentration well below the 50% inhibitory concentration (IC_50_) for this strain (3), AE104 cells accumulate 120,000 zinc atoms per cell, and the efflux systems are required to prevent a further increase of the zinc content (11). The pleiotropic phenotype of the Δ*zupT* mutant, however, is not completely restored to the level of the parent strain by addition of external zinc (10, 11). This demonstrates that different zinc pools and zinc delivery channels exist in the *C. metallidurans* cell.

It can also be expected that subsequent deletion of one metal importer after the other should finally lead to a lethal phenotype or to cells in need of high external metal concentrations. This point was not reached with the Δ7 mutant (Δ*zupT ΔpitA ΔcorA*_1_ *ΔcorA*_2_ *ΔcorA*_3_ *ΔzntB ΔhoxN*); however, while deletion of transporter genes up to the preceding Δ6 mutant (Δ*zupT ΔpitA ΔcorA*_1_ *ΔcorA*_2_ *ΔcorA*_3_ *ΔzntB*) kept metal homoeostasis and cellular fitness of the Δ6 mutant at the level of the Δ*zupT* single mutant, additional deletion of *hoxN* in Δ6, leading to Δ7, decreased both features to another level (7). During the course of the construction of the multiple-deletion mutants, no upregulation of genes for metal importers could be observed, with the exception of *hoxN* in Δ6, suggesting that there are no “reserve” genes in *C. metallidurans* that are silent in strain AE104 but upregulated in deletion mutants to compensate for the respective absent importer. Instead, (with the exception of HoxN) all metal importers must be present in all strains from the parent AE104 to the Δ7 strain. Loss of importers in each mutant strain always could be compensated for by the respective remaining transporters. This high plasticity of the metal uptake transportome allows the mutants up to Δ6 to remain at the fitness level of the Δ*zupT* single mutant strain, but in Δ7, this plasticity is diminished (7). This paper starts with a transcriptome and morphology analysis of Δ7 to investigate the reason for the decreased fitness of this strain, which seems to result from a negative interference between the action of two metal-transporting P-type ATPases.

## RESULTS

### Cellular morphology of Δ7 strain.

**FIG 1:**
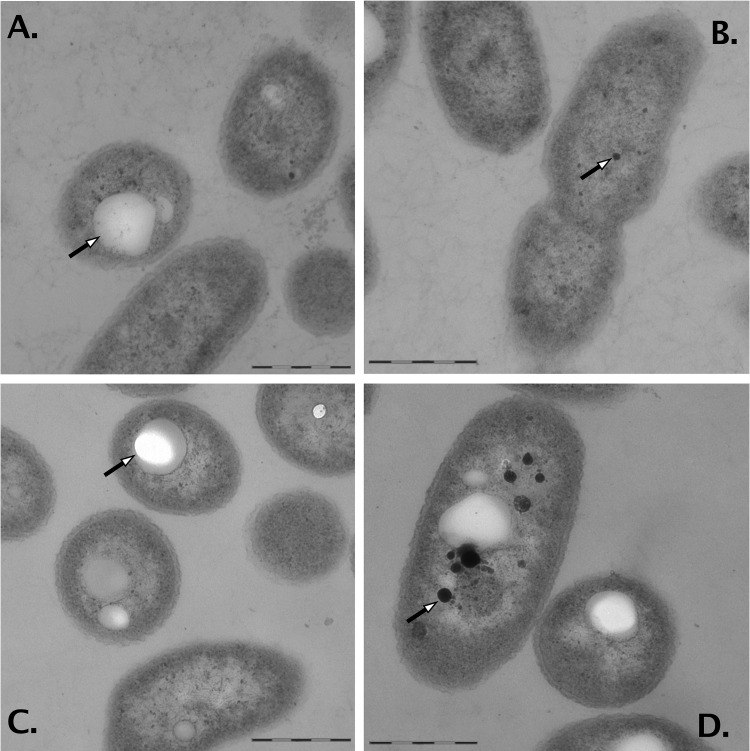
Cellular morphology of *C. metallidurans* strain Δ7 and its parent strain AE104. Parent strain AE104 (A and B) and strain Δ7 (C and D) were cultivated in TMM. Thirty-one percent of the AE104 cell sections (*n* = 85) contained grayish particles; three cell sections contained one large particle that filled the section to a large extent (A, arrow). AE104 cell sections contained only small single electron-dense particles (B, arrow). The number of Δ7 cell sections containing grayish particles was similar to that of the AE104 cells (28%, *n* = 118). Differences between the AE104 and Δ7 particles were most likely preparation artifacts (A and C, arrows). An 8.5% proportion of the Δ7 cell sections contained one or more electron-dense particles (D, arrow) that displayed a higher electron density and a larger diameter than the electron-dense particles in strain AE104 (B, arrow). Bars, 0.3 µm (D) or 0.5 µM (A to C).

The Δ7 strain and its parent strain AE104 were cultivated in Tris-buffered mineral salts medium (TMM), which contains transition metals only at trace element (nanomolar) concentrations (13). The Δ7 and AE104 cell sections were not different in cell dimensions and general morphology (Fig. 1). About 30% of both kinds of cell sections contained grayish particles probably representing poly-beta-hydroxic acid, a known storage compound in *Cupriavidus* species (14). A few cell sections contained large representatives of these particles that nearly filled the whole cell (Fig. 1A and C). An 8.5% proportion of the Δ7 cell sections contained one or more electron-dense particles (Fig. 1D) that were fewer, smaller, and less electron dense in the parent strain (compare Fig. 1B and D). The respective particles in AE104 cell sections contained a high phosphate content (N. Wiesemann and D. H. Nies, unpublished data).

### Transcriptome of Δ7.

The global transcriptome of Δ7 was analyzed in cells grown under global metal starvation conditions (50 µM EDTA) or in the presence of sufficient zinc (10 µM zinc chloride). The Tris-buffered mineral salts medium (TMM) used (13) contains 1 mM Mg(II), 0.2 mM Ca(II), 4.3 µM iron ammonium citrate, and nanomolar concentrations of all other essential transition metals plus 642 µM phosphate. These data were also compared to those of the Δ*zupT* strain and the AE104 parent (Table 1; also see Table S1 in the supplemental material). All values with an up- or downregulation more than 2-fold (*Q* of ≤0.5 or ≥2.0) were considered. Namely, nearly 10% of the 5,255 genes analyzed changed expression, 307 in the comparison of Δ7 and Δ*zupT* strains, 144 in the comparison of Δ7 and AE104 strains, and another 144 in AE104 in the comparison of EDTA and Zn(II) (Table 1). Differences were judged as significant if their *D* values were larger than 1, meaning that the deviation bars of the respective data points did not touch or overlap. Three biological reproductions were done for Δ7 and AE104, and two were done for the Δ*zupT* strain. A comparison of the Δ*zupT* strain to AE104 has been published elsewhere (12). All genes located in an uninterrupted series in the same direction of transcription had been previously numbered and designated “operon regions” (12) to facilitate analysis of the *C. metallidurans* genome and its transcription, “f” for forward direction and “r” for reverse.

10.1128/mSystems.00004-16.1 Table S1  Comparison of the transcriptomes of the AE104, Δ*zupT*, and Δ7 strains. For the gene array experiment, strain Δ7, the Δ*zupT* mutant, and the AE104 parent were cultivated in TMM including 50 µM EDTA or 10 µM Zn(II). RNA was isolated, reverse transcribed and used in a gene array experiment. Three biological replicates were done for AE104 and Δ7, and two were done for the Δ*zupT* mutant. *Q* ratios of the mean values and *D* values are given. Differences were counted as significant if *Q* was >2 or <0.5 and *D* was >1. Upregulated *Q* values are in green; downregulated *Q* values are in red; *D* values of >1 are in bold; *D* values of ≤1 are in italic. Protein numbers per cell are from reference 11. NeF, never found; NF, not found in the respective strain; NQ, could not be quantified. Download Table S1, PDF file, 0.2 MB. Copyright © 2016 Große et al. 2016 Große et al. This content is distributed under the terms of the Creative Commons Attribution 4.0 International license.

**TABLE 1:**
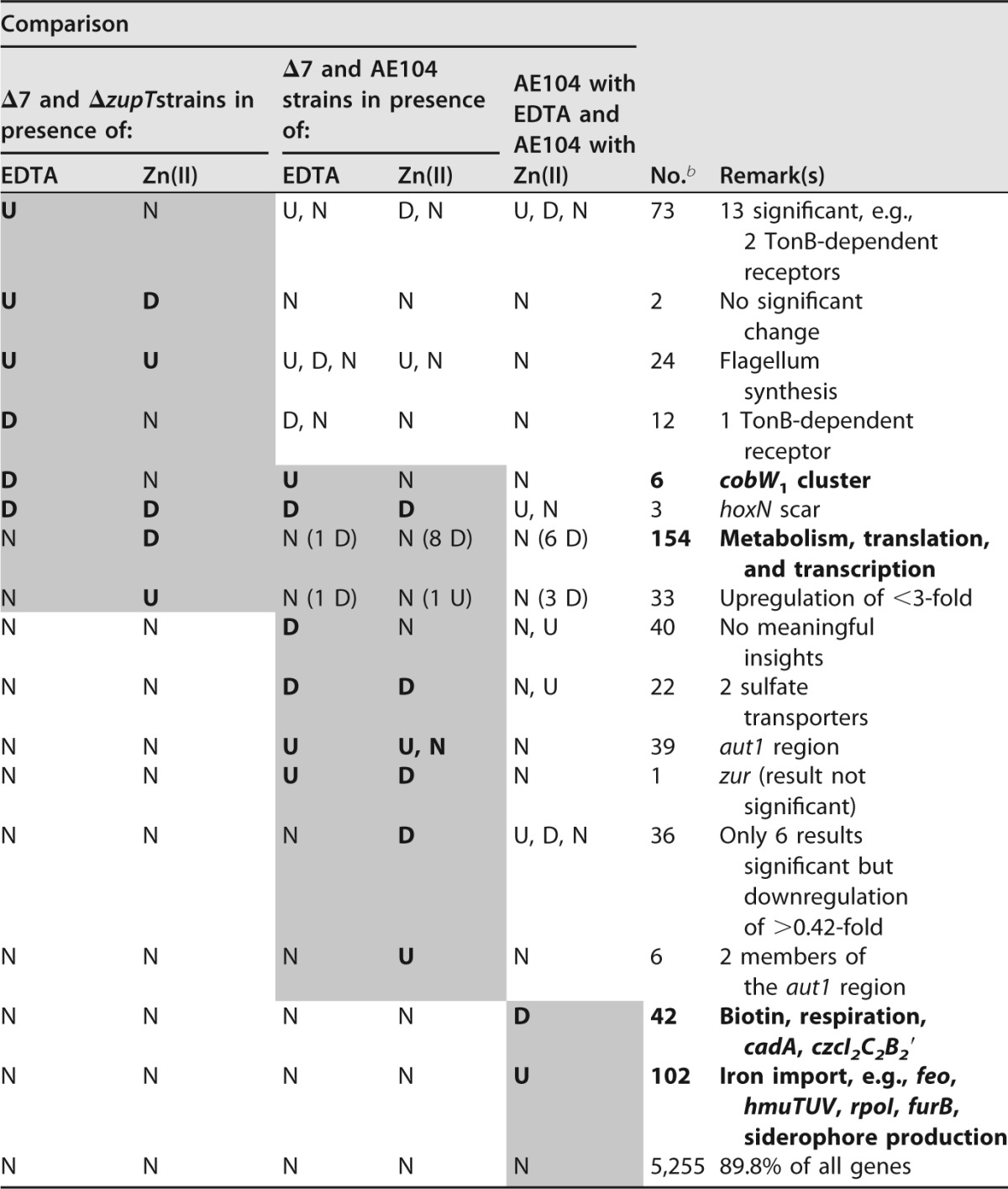
Changes in global transcriptome of Δ7 mutant compared to *zupT* single mutant and parent strain AE104^a^

a Regulation of 5,850 chromosomal genes in the comparison (quotient of the mean signals *Q*) of Δ7 and Δ*zupT* strains and Δ7 and AE104 strains in the presence of 50 µM EDTA or 10 µM Zn(II). The comparison of these conditions in the parent strain is indicated as upregulated (U, *Q* ≥ 2), downregulated (D, *Q* ≤ 0.5), or not regulated (N). The full results are given in Table S1 and elsewhere in the supplemental material. Shaded areas indicate the differences used to sort the data into groups. Boldfaced letters highlight the most important results.

b All genes regulated in the indicated pattern, including nonsignificant results (*D* < 1).

### Comparison of Δ7 and Δ*zupT* strains.

Half of the genes (154 of 307) that were changed in expression in the comparison of Δ7 and Δ*zupT* strains were downregulated by zinc but not regulated by EDTA, agreeing with the lower zinc resistance of the Δ7 strain than of the Δ*zupT* strain (7). These genes encoded ribosomal proteins and translation factors; Sec- and TAT-dependent protein export; the components of the RNA polymerase; the HU DNA-binding protein Rmet_4749 and other histone-like, DNA- and small RNA-binding proteins; FtsZ; the components of the F_1_F_0_ ATPase; sulfate adenylyltransferase; components of the tricarbonic acid cycle; phosphoglycerate mutase; and glyceraldehyde-3-phosphate dehydrogenase. Deletion of the other 6 metal import systems leading from the Δ*zupT* strain to the Δ7 mutant resulted in a global zinc-dependent downregulation of the overall metabolic backbone. Zinc exporters such as ZntA (3, 15) were not upregulated, and the zinc concentration used was very low (10 µM), so that this global zinc-dependent response was more likely a regulatory event than the result of excessive zinc stress.

In agreement with this, six out of the 12 genes that were downregulated in the comparison of Δ7 and Δ*zupT* strains in EDTA but not by zinc were the genes in the *cobW*_1_-cluster zinc starvation cluster Op0317f (mean ratio, 0.29- ± 0.02-fold), which were strongly upregulated in the presence of EDTA in the Δ7-AE104 comparison (7.46- ± 1.50-fold) but only slightly upregulated in AE104 under zinc starvation (1.75- ± 0.37-fold). Regulation of expression of the *cobW*_1_ gene Rmet_1098 has been published previously (12) and was again confirmed by reverse transcription-PCR (RT-PCR) (Table 2) and *lacZ* fusions (see Table S2 in the supplemental material), here serving as a positive control for zinc starvation conditions. Transcription of *cobW*_1_ was induced by 50 µM EDTA (Table 2), more strongly in the Δ*zupT* strain than in Δ7, and more strongly in both strains than in the parent AE104. Since the *cobW*_1_ cluster was upregulated only under severe zinc starvation conditions, this indicated that zinc starvation in the Δ7 strain was reduced compared to that in the Δ*zupT* strain.

10.1128/mSystems.00004-16.2 Table S2  Regulation of *lacZ* fusions with genes that might be involved in zinc homeostasis. The values below the gene names are the specific activities in AE104 cells cultivated in TMM without further additions. All other values were divided by this basic activity. Red bold numbers indicate significant (*D* > 1) downregulation; green bold numbers indicate significant upregulation. Download Table S2, PDF file, 0.1 MB. Copyright © 2016 Große et al. 2016 Große et al. This content is distributed under the terms of the Creative Commons Attribution 4.0 International license.

**TABLE 2:** RT-PCR verification of gene array data^a^

Gene	Signal for strain in presence of Zn or EDTA					
	Δ7		Δ*zupT* mutant		AE104	
	Zn	EDTA	Zn	EDTA	Zn	EDTA
*rpoZ* (control)	++	++	++	++	++	++
Rmet_1098, *cobW*_1_	/	++	/	+++	/	+
Rmet_0837	/	++	/	++	/	+(+)
Rmet_5640	/	(+)	/	/	/	(+)
Rmet_5890, *feoB*	/	+(+)	+	++	+	+(+)
Rmet_1533, *hoxN*	/	/	++	++	(+)	(+)
Rmet_1794	/	/	/	/	+	(+)

a To cross-check some gene array data points with an independent method, the presence of the mRNAs for six genes was semiquantified using RT-PCR. Cells of AE104, Δ*zupT*, and Δ7 strains were incubated in TMM in the presence of 10 µM Zn(II) or 50 µM EDTA, and RNA was isolated, reverse transcribed, and amplified by PCR. Three experiments with a positive control of DNA and a negative control with water were performed. /, no signal; other symbols represent signal strength decreasing from +++ and ++ via +(+) and + to (+), which represents a weak signal.

Thirteen genes were significantly upregulated in the presence of EDTA but not changed in expression by zinc (Table 1); 10 of them are involved in chemotaxis, one encodes a putative uncharacterized protein, and two encode a TonB-dependent receptor and an uncharacterized protein adjacently encoded. Twenty-four genes were upregulated in the comparison of Δ7 and Δ*zupT* strains in the presence of EDTA and of zinc. Genes encoded flagellum synthesis and an ABC importer, Rmet_3185/86, in Op0895f. These two genes were also upregulated in the Δ7-AE104 comparison independently of the conditions but not by metal starvation in strain AE104 (see Table S1 in the supplemental material). No other gene encoding an uncharacterized membrane protein or a putative transporter was upregulated by EDTA specifically in the Δ7 strain.

Taken together, the Δ7 strain managed to ameliorate its zinc starvation condition to some extent but at the cost of an increased zinc sensitivity of its metabolic backbone. This happened despite the presence of powerful zinc efflux systems such as ZntA and CadA, which were not changed in expression in Δ7 compared to the Δ*zupT* strain. Moreover, no “reserve” genes for additional metal uptake systems were upregulated in Δ7 compared to the Δ*zupT* strain, although Δ7 managed to import all essential metals despite the deletion of seven transporters.

### Comparison of Δ7 and AE104.

A total of 144 genes were unchanged in expression in the comparison of Δ7 and Δ*zupT* strains but were altered in the Δ7-AE104 comparison; however, genes for metal transporters were not among them (Table 1). Thirty-eight genes of the *aut1* region (plus 1 more gene outside the *aut1* region) encoding the soluble hydrogenase and Calvin cycle proteins were upregulated in the Δ7-AE104 comparison independently of the growth conditions, confirming the published unsilencing of the *aut1* region as a result of the Δ*zupT* deletion (12). Additional deletion of metal uptake systems did not change expression of *aut1* again. Other results of the Δ7-AE104 comparison did not lead to further insights.

### Metal starvation versus zinc supply in the parent strain AE104.

A total of 42 genes were downregulated in the EDTA-Zn(II) comparison in the parent strain AE104 (Table 1). These genes encoded components of the tricarbonic acid cycle (TCC), the iron-containing superoxide dismutase SodB, the elongation factor Ts, biotin synthesis, the cadmium/zinc-exporting P_IB_-type ATPase CadA (but not ZntA), and the *czcI_2_C_2_B_2_*′ cluster for the interrupted *czc*-like system on chromosome 2. While downregulation of SodB and the TCC proteins indicated iron starvation, that of CadA and the ancient chromosomal *czc* system (4) indicated an EDTA-mediated decrease of the zinc stress in strain AE104 (12).

A greater number of genes, 102, were upregulated in AE104 in the EDTA-Zn(II) comparison. These encoded many proteins involved in iron uptake such as the siderophore biosynthesis region, TonB-dependent receptors, the ECF sigma factors RpoI and RpoK, the regulator FurB but not FurA, the Feo system, and the HmuTUV ABC-type importer. The *feoB* gene was upregulated in the presence of EDTA in the AE104, Δ*zupT*, and Δ7 strains as indicated by RT-PCR experiments (Table 2) and the HmuTUV transporter also as shown with *lacZ* fusions, but also by addition of metal cations, except in the Δ7 strain (see Table S2 in the supplemental material).

This indicated that treatment of strain AE104 with EDTA decreased zinc stress but yielded iron starvation, which was compensated for by upregulation of siderophore-dependent and alternative iron uptake pathways. Due to the presence of ZupT in AE104 cells, there was no zinc starvation in AE104 as indicated by the strong upregulation of the *cobW*_1_ zinc starvation gene cluster in EDTA-treated Δ*zupT* cells compared to AE104 cells (12).

The gene *zntA* for the mainly zinc-exporting P_IB_-type ATPase (3, 15), however, was unchanged in all of the strains and under all of the conditions tested, indicating that no increased zinc stress occurred during these experiments. No changes in expression under any of these conditions were observed for Rmet_5396 (*mgtA*) and Rmet_2211 (*mgtB*), both encoding P-type ATPases that might be involved in magnesium transport (see the supplemental material). Other genes possibly involved in metal transport were also not changed in expression: Rmet_0450 (*corC*), Rmet_4765, Rmet_1762, and Rmet_0698 (*corC-*like).

Thus, the unknown metal importers supplying metals to the Δ7 cells were not the products of some “reserve” genes activated in this multiple mutant strain. Instead, and starting from the AE104 parent strain, all mutant derivatives contained more components of the metal uptake transportome than the seven secondary importers, and these systems were able to take over when one uptake system after the other was removed from the cells. The residual transportome working in Δ7, however, displayed a diminished competence to maintain metal homoeostasis as indicated by the increased metal sensitivity and zinc-dependent downregulation of the core metabolic processes.

### Reporter gene fusions.

To screen for additional components of the metal uptake transportome, regulation of expression of some genes noticeable in the transcriptome analysis was characterized in more detail using transcriptional *lacZ* reporter gene fusions. The *lacZ* reporter gene was inserted within several target genes to construct operon fusions. Of the disruption strains constructed (see Table S2 in the supplemental material), only Rmet_1794, Rmet_1819, and Rmet_5377 (*hmuU*) showed a change in metal resistance, but only in the Δ*zupT* mutant background (data not shown). To test expression of these and other genes, similar gene disruption strains in AE104 and Δ*zupT* strain backgrounds were constructed and compared to expression of *cobW*_1_ (Rmet_1098) in these strains as an indicator of zinc starvation. The tested strains (see Table S2) confirmed upregulation by EDTA in the cases of Rmet_0837, the positive-control Rmet_1098, Rmet_1106, Rmet_1114, Rmet_1819, Rmet_5377, Rmet_5460, and Rmet_5747 but not Rmet_1794. The upregulated genes may contribute to metal homoeostasis.

**FIG 2:**
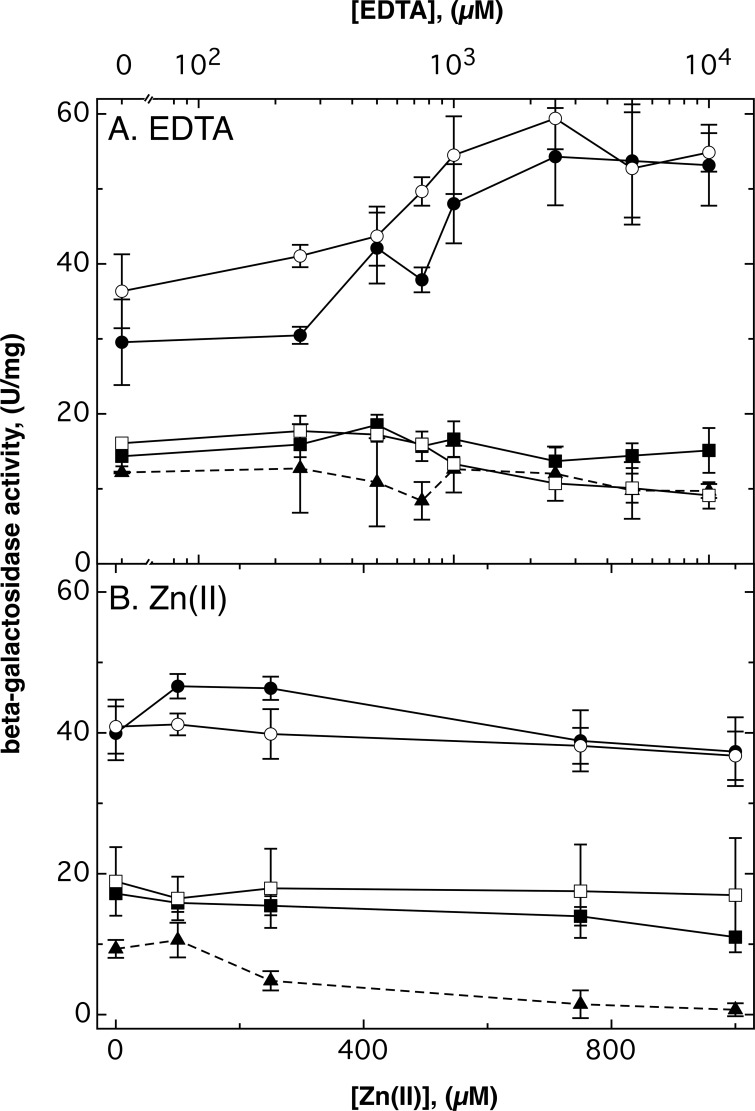
Regulation of *mgtA-lacZ* and *mgtB-lacZ* transcriptional fusions in *C. metallidurans*. Exponentially growing cells of strain AE104 (closed circles or squares), the Δ5 mutant (open circles or squares), and the Δ7 mutant (closed triangles) carrying a *lacZ* fusion inside and interrupting the *mgtA* (circles and triangles) or *mgtB* (squares) genes were divided into parallel cultures, and EDTA (A) or zinc chloride (B) was added. Incubation was continued with shaking at 30°C, and the specific activity of β-galactosidase was determined after 3 h (*n* ≥ 3). Standard deviations are shown as bars.

In a similar approach, the *mgtA* (Rmet_5396) and *mgtB* (Rmet_2211) genes, encoding P-type ATPases possibly involved in calcium or magnesium transport, were also disrupted in strain AE104. The basic expression level of the *mgtA-lacZ* fusion was about two times higher than that of the *mgtB* fusion (Fig. 2). The genes were not regulated by zinc (Fig. 2). No difference measured was significant regardless of whether the genes were interrupted (as in Fig. 2) or whether a full-length fusion of the gene with *lacZ* was constructed (data not shown). High EDTA concentrations (>250 µM), however, increased the expression level of *mgtA-lacZ* but not that of *mgtB-lacZ* (Fig. 2). When the *mgtA-lacZ* fusion was constructed in the Δ7 mutant strain, the expression level was decreased and no longer regulated by EDTA. Instead, increasing zinc concentrations downregulated *mgtA-lacZ* expression from 9.33 ± 1.27 U/mg dry mass at no added zinc to 0.69 ± 0.92 U/mg (representing no activity at all in two of the three repetitions) at 1 mM Zn(II) (Fig. 2). Similar fusions were constructed in the Δ5 mutant strain, but there was no difference compared to strain AE104 (Fig. 2). Loss of *zntB* and *hoxN* in the Δ5 strain, leading to the Δ7 strain, changed the expression pattern of *mgtA*, indicating that its gene product was recruited for metal uptake.

### Deletion of the genes for putative magnesium-importing P-type ATPases.

The gene *mgtA* was interrupted in the Δ7 strain (Δ7A, Δ7 Δ*mgtA*::*kan*), and *mgtB* was deleted, leading to the Δ8 multiple deletion mutant. In a last step, *mgtA* was disrupted in Δ8 by insertion of a kanamycin resistance cassette, leading to Δ9. The metal contents of Δ8 and Δ9 were similar to that of the Δ7 strain, with the exception of the magnesium content, which was significantly lower in the Δ9 strain than in the Δ7 strain but not at the low level of strain AE104 (Table 3). Net zinc, magnesium, or general transition metal import was not abolished in the Δ9 strain. MgtA and/or MgtB was not essential for Mg import in *C. metallidurans*, although the P-type ATPases contributed to the high Mg content of strain Δ7.

**TABLE 3:** Metal content of *C. metallidurans* mutant strains^a^

Bacterial strain	No. of metal atoms per cell					
	Mg, 10^6^	Fe, 10^3^	Zn, 10^3^	Cu, 10^3^	Co, 10^3^	Ni, 10^3^
AE104^b^	11.5 ± 1.1	743 ± 70	72.8 ± 9.5	9.00 ± 1.53	3.32 ± 0.71	4.68 ± 2.21
Δ*zupT*^b^	9.9 ± 1.2	708 ± 106	21.1 ± 5.8	10.97 ± 3.39	5.92 ± 1.58	3.07 ± 0.35
Δ7 (Δ6 Δ*hoxN*)^b^	43.5 ± 3.9	745 ± 59	31.6 ± 3.9	13.43 ± 3.83	5.19 ± 1.35	1.48 ± 0.30
Δ8 (Δ7 Δ*mgtB*)	39.8 ± 6.2	693 ± 130	31.8 ± 7.2	13.33 ± 2.34	5.38 ± 1.16	1.71 ± 1.46
Δ9 (Δ8 Δ*mgtA*::*kan*)	30.4 ± 5.1	553 ± 118	23.2 ± 4.5	10.17 ± 2.21	4.20 ± 1.17	1.30 ± 0.93

a The bacterial strains were cultivated in TMM, and the metal content was determined by ICP-MS. Some values are additional reproductions under parallel conditions with the new mutant strain.

b Previously published values (2, 7, 10) for AE104, Δ*zupT*, and Δ7 strains were provided for reference.

### Growth of the mutant strains in liquid TMM in the presence and absence of transition metal cations.

**FIG 3:**
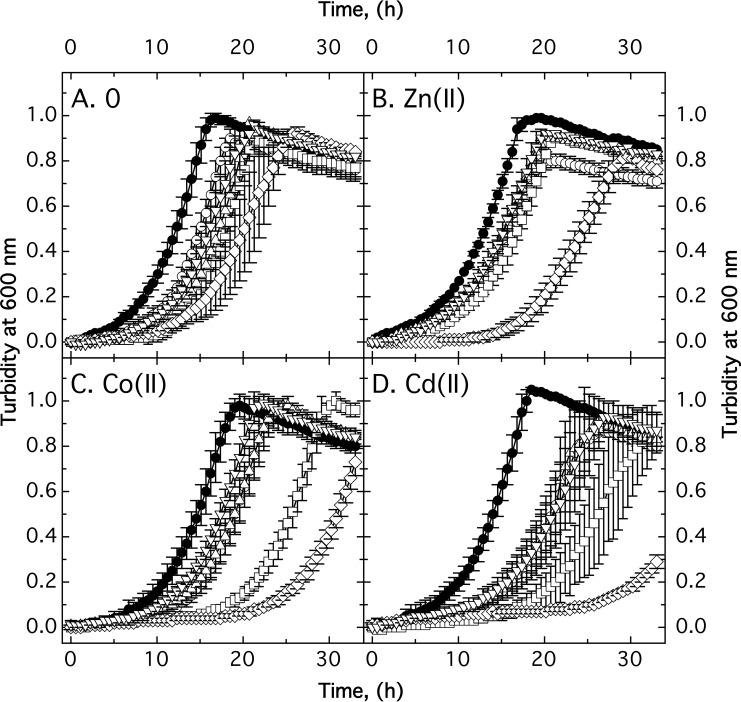
Growth impairment of the mutant strains. The AE104 (closed circles), Δ*zupT* (open circles), Δ7 (open squares), Δ8 (open triangles), Δ7 *mgtA*::*kan* (open inverted triangles), and Δ9 (open diamonds) strains were cultivated in TMM at 30°C without further additions (A) or in the presence of 10 µM Zn(II) (B), 10 µM Co(II) (C), or 2.5 µM Cd(II) (D), and growth was measured as turbidity at 600 nm (*n* ≥ 3). Standard deviations are indicated by bars.

Growth of the Δ7 strain with all known secondary zinc uptake systems deleted was retarded in nonamended TMM compared to the Δ*zupT* strain and parent AE104. Addition of a low zinc concentration (10 µM) compensated for this growth defect partially, while 10 µM Co(II) and especially 2.5 µM Cd(II) increased it (7) (Fig. 3). Deletion of *mgtB* or disruption of *mgtA* in strain Δ7 improved growth again under all four conditions (Fig. 3), also in the presence of 250 µM Cu(II) and 50 µM EDTA (data not shown). Both strains grew similarly to the Δ*zupT* deletion strain. Deletion of both genes, however, severely affected the cells, even when 10 µM Zn(II) was added, which rescued all other mutations up to Δ8 and Δ7A to the Δ*zupT* level of fitness (Fig. 3).

The growth rates of the cells were not affected in nonamended medium or in the presence of 10 µM Co(II) or 10 µM Zn(II) (Table 4) and remained between 0.2 h^−1^ and 0.3 h^−1^, representing a doubling time between 2.3 and 3.5 h. While the growth rate of strain AE104 cultivated in the presence of 2.5 µM Cd(II) was not different from that of strain AE104 cultivated without added cadmium, the growth rate in the presence of this metal decreased slightly, but not significantly, with an increasing number of deletions from 0.23 ± 0.06 h^−1^ to 0.17 ± 0.04 h^−1^ in the Δ8 mutant (Table 4). The duration of the lag phase increased in the Δ*zupT* mutant and partially the Δ7 strain compared to strain AE104 in nonamended medium, in the presence of 10 µM Co(II) or 2.5 µM Cd(II) but not in the presence of zinc. It decreased again, when the *mgtA* or the *mgtB* gene was deleted (Table 4).

**TABLE 4:** Growth parameters of the mutant strains^a^

Bacterial strain	Growth rate (1/h)/duration of the lag phase (h) after addition of metal:			
	None	10 µM CoCl_2_	10 µM ZnCl_2_	2.5 µM CdCl_2_
AE104	0.29 ± 0.03/0	0.26 ± 0.07/0	0.23 ± 0.01/0	0.23 ± 0.06/0
Δ*zupT*	0.28 ± 0.06/1.0	0.26 ± 0.03/3.9	0.22 ± 0.04/0	0.22 ± 0.06/2.8
Δ7	0.34 ± 0.15/6.1	0.21 ± 0.02/7.0	0.23 ± 0.01/0	0.20 ± 0.07/6.2
Δ7 *mgtA*::*kan*	0.26 ± 0.02/0.9	0.23 ± 0.06/0.5	0.24 ± 0.03/0	0.16 ± 0.04/0
Δ8	0.29 ± 0.00/3.3	0.22 ± 0.03/1.0	0.21 ± 0.01/0	0.17 ± 0.04/0
Δ9	0.23 ± 0.05/2.8	0.19 ± 0.06/10.9	0.27 ± 0.08/9.0	0.06 ± 0.01/0

a Strain AE104 and its mutant derivatives were cultivated in TMM at 30°C without further additions or in the presence of added metal salts, and growth was measured as turbidity at 600 nm (Fig. 3). The mean data points of 3 reproductions were used to calculate the growth rate and duration of the lag phase.

**FIG 4:**
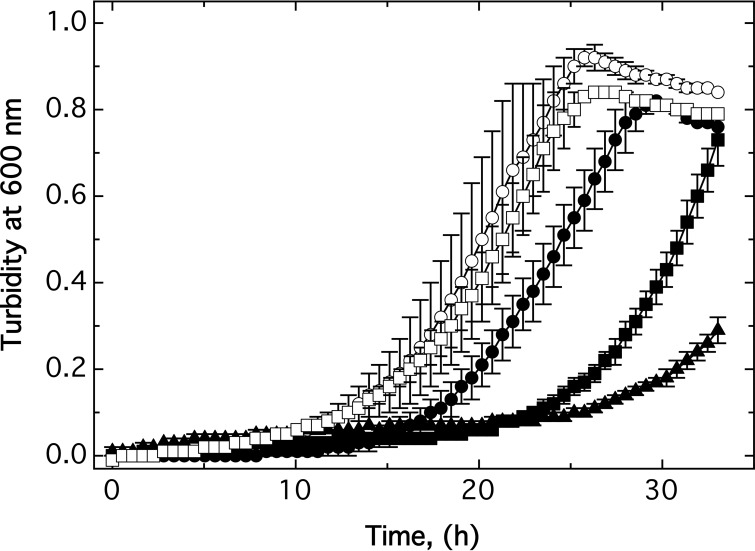
Growth impairment of the Δ9 mutant strain. The Δ9 mutant strain was cultivated in TMM at 30°C without further additions (open circles) or in the presence of 50 µM EDTA (open squares), 10 µM Zn(II) (closed circles), 10 µM Co(II) (closed squares), or 2.5 µM Cd(II) (closed triangles), and growth was measured as turbidity at 600 nm (*n* ≥ 3). Standard deviations are indicated by bars.

Growth of the Δ9 mutant strain was compromised in the absence of added metal and even more by addition of 10 µM Zn(II) ≪ 10 µM Co(II) ≪ 2.5 µM Cd(II), while addition of 50 µM EDTA had only a small effect (Fig. 4). While the duration of the lag phase of cells cultivated in nonamended medium remained at the length of that of the Δ8 strain (Table 4), the lag phase was extended in cobalt- and zinc-cultivated cells. In the presence of cadmium, the growth rate was decreased to 0.06 h^−1^, representing a doubling time of nearly 12 h. The lower level of metal and oxidative stress resistance, especially of the Δ9 strain, could also be confirmed in liquid culture (Table 5).

**TABLE 5:** Resistance of deletion strains in liquid culture^a^

Bacterial strain	IC_50_ (µM) of agent:								
	Zinc	Cobalt	Cadmium	Copper	Gold^b^	Nickel	EDTA	H_2_O_2_	Paraquat
AE104 parent strain	**442 ± 40 **	**94 ± 6**	**93 ± 10**	**906 ± 82**	**25.3 ± 2.1**	**348 ± 16**	249 ± 9	**2,650 ± 279**	169 ± 10
AE104 Δ*zupT*	**156 ± 16**	**34 ± 6**	**16 ± 3**	**400 ± 77**	13.0 ± 1.3	171 ± 10	183 ± 13	894 ± 133	111 ± 6
Δ7	53 ± 4	8.3 ± 1.6	2.19 ± 0.4	265 ± 32	14.8 ± 2.6	145 ± 16	205 ± 22	989 ± 102	135 ± 4
Δ7 Δ*mgtA*::*kan*	**113 ± 8**	**43 ± 8**	**8.5 ± 0.8**	**449 ± 51**	13.2 ± 2.4	**352 ± 16**	217 ± 32	ND^c^	ND
Δ8 (=Δ7Δ*mgtB*)	**145 ± 9**	**45 ± 7**	**9.0 ± 0.7**	**444 ± 46**	9.5 ± 2.4	**367 ± 12**	210 ± 11	**421 ± 75**	**64 ± 5**
Δ9 (=Δ8Δ*mgtA*::*kan*)	**176 ± 27**	**3.7 ± 0.5**	**0.40 ± 0.04**	**681 ± 72**	**25.8 ± 5.2**	ND	**406 ± 43**	**491 ± 96**	**71 ± 10**

a Dose-response experiments were performed (*n* > 3 per condition), and the IC_50_ values were calculated. Boldface values indicate significant deviations from metal resistance of Δ7.

b Au(III)Cl_4_^−^. The values for AE104, Δ*zupT*, and Δ7 strains have been published elsewhere (7) and are given for reference.

c ND, not done.

The extended lag phase determined in many experiments was not likely the result of a subpopulation of suppressor mutants. The longest lag phase measured was 10.9 h in the case of the Δ9 strain in the presence of cobalt (Table 4). These cells grew at a rate of 0.19 h^−1^ or a doubling time of 3.65 h, indicating that three duplications could have been accomplished. Consequently, the ratio of a suppressor mutant subpopulation must have been 12.5%, which is unlikely. Instead, and at least for the Δ8 mutant strain, the lack of affected growth rates, the extended lag phases in the presence of cobalt and cadmium, and the reversal of these effects in the presence of 10 µM Zn(II) all indicated that these strains had increasing problems in adjusting their zinc homeostasis during the lag phase as a prerequisite for growth and that this adjustment was disturbed by cobalt and cadmium. In the case of the Δ9 mutant, a new lower level was reached, and now even the addition of zinc could no longer recover the disturbed metal homeostasis in this mutant strain.

Taken together, a negative interference between the actions of MgtA and MgtB was responsible for the decreased fitness of the Δ7 strain, visible as zinc-dependent downregulation of the overall core metabolic processes. On the other hand, both P-type ATPases kept Δ7 on a fitness level above that of the Δ9 strain, clearly indicating the contribution of MgtA and MgtB to fulfilling backup roles in metal supply in *C. metallidurans*.

## DISCUSSION

The seven secondary metal uptake systems ZupT, PitA, CorA_1_ to CorA_3_, ZntB, and HoxN seem to interact and form together with the metal efflux systems ZntA, CadA, DmeF, and FieF the core of the metal transportome of *C. metallidurans* (7). The metal uptake transportome shows much plasticity, since deletion of most importers could be compensated for in most part by activation of other importers with subsiding specificity function. The exception was the Δ*zupT* deletion, which resulted in a decreased cellular zinc level and many other pleiotropic effects (10–12).

With an increasing number of deletions in metal import systems, the *C. metallidurans* cells, when grown in nonamended TMM, remained at the fitness level and overall metal content of the Δ*zupT* mutant strain, although especially the cobalt and cadmium resistance of the multiple deletion mutants dropped with each added deletion. The cells could tolerate deletion of the above-mentioned “triad” PitA-CorA proteins plus ZupT and even an additional deletion of the zinc transporter ZntB. When, however, the remaining secondary metal import system HoxN, proposed to supply nickel ions to the hydrogenases of the bacterium, was removed, fitness and Co(II)/Cd(II) sensitivity of the resulting Δ7 mutant reached a new minimum (7).

The cytoplasm of Δ7 contained a high number of electron-dense particles (Fig. 1). Similar, but smaller, particles in strain AE104 were polyphosphate granules (Wiesemann and Nies, unpublished), so that the increased electron density might indicate binding of surplus magnesium to polyphosphate granules. Together, these data indicate that the disturbed transition metal homeostasis in combination with the resultant higher magnesium content affected cell physiology, as has also been observed in *Haemophilus influenzae* (16).

The Δ7 strain could maintain its overall cellular metal content with the exception of a lower zinc and higher magnesium level at the cost of increased cobalt, cadmium, and zinc sensitivity (7). The latter resulted from a higher impact of zinc toxicity on the central metabolism of the cells despite signs of zinc starvation at the same time. No clear upregulated candidate genes were identified as being obvious candidates in the Δ7 strain that were responsible for metal supply in this strain, indicating unknown members of the metal uptake transportome composed of importers already in place in the parent strain AE104. This suggests either posttranscriptional regulation of preexisting proteins or posttranslational activation of existing transporters.

**FIG 5:**
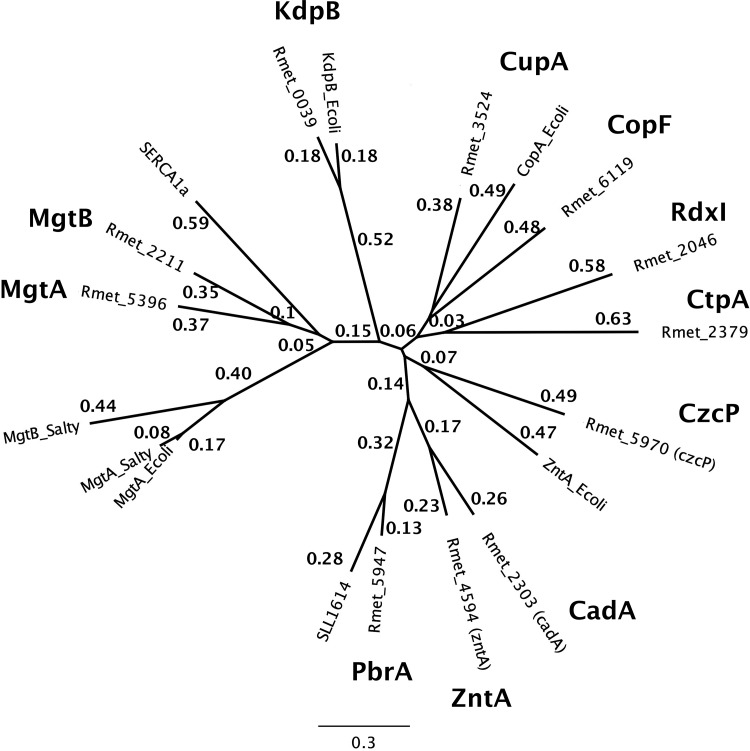
Relationship of the P-type ATPases from *C. metallidurans*. A multiple alignment of all P-type ATPases from *C. metallidurans* with all *E. coli* proteins, MgtA and MgtB from *Salmonella*, SERCA1a from *Rattus norvegicus*, and SLL1614 from the cyanobacterium *Synechocystis* was performed, showing the position of the three P_IB2_-type ATPases for export of lead (PbrA), zinc (ZntA), and cadmium (CadA); the P_IB4_-type zinc exporter CzcP; the anabolic (CtpA and RdxI) and detoxifying (CopF and CupA) copper exporter; the part of the potassium/sodium exchange system KdpB; and finally Rmet_2211 (MgtB) and Rmet_5396 (MgtA), which were much more closely related to SERCA1a than to MgtA and MgtB from *Salmonella*. The alignment was constructed with Geneious 6.1.6 (Biomatters Ltd., Auckland, New Zealand) using the Blosum 62 cost matrix with an open gap penalty of 12 and a gap extension penalty of 3, the Jukes-Cantor genetic distance model, and the neighbor-joining tree building method. Numbers of substitutions per site are indicated, along with a scale bar.

The two P-type ATPases MgtA (Rmet_5396) and MgtB (Rmet_2211), annotated as Mg^2+^/Ca^2+^ importers (Fig. 5), were identified as two of these proteins that have altered metal specificity in the Δ7 background. Deletion of the respective genes resulted in a decreased magnesium content of the Δ9 mutant compared to the Δ7 cells, indicating that these proteins are involved in magnesium transport. Moreover, deletion of these genes yielded further loss of fitness, and Co(II)/Cd(II) resistance decreased in the resulting mutant strain Δ9 (Fig. 3 and 4). Interestingly, single gene deletion or disruption of either gene in Δ7 increased fitness and Co(II)/Cd(II) resistance again, indicating that the presence of either P-type ATPase negatively interfered with the other protein, similar to the negative interference of PitA and the CorA proteins in the Δ*zupT* mutant strain when it comes to an efficient allocation of zinc to RpoC. The Δ9 mutant had more problems than the Δ7 mutant, indicating that both P-type ATPases increased performance of the Δ7 mutant, but surprisingly, either one of these systems was even better than the two together.

The Δ9 mutant was still able to maintain its cellular metal content, indicating the presence of at least one more metal import system for Mg(II), Zn(II), and other metals, and the activity of this system should be responsible for the low Co(II)/Cd(II) resistance of the Δ9 mutant, despite the presence of the cadmium efflux system CadA and the cobalt efflux system DmeF in Δ9. *Escherichia coli* and *Salmonella* do not contain such an additional metal uptake system; they need higher magnesium concentrations in the growth medium when CorA and MgtA (*E. coli*) or CorA, MgtA, and MgtB (*Salmonella*) are deleted (17–20), and they no longer import magnesium. The two MgtA and MgtB proteins from *C. metallidurans* are much more closely related to the Ca(II)-transporting SERCA protein from muscles than to the Mg(II) importers from *Salmonella* (Fig. 5). This means that MgtA and MgtB from *C. metallidurans* may have a function different from that of the respective proteins in *Salmonella*. Nevertheless, the decrease of the cellular magnesium content and the change in fitness and metal resistance upon deletion of the two genes in Δ7 clearly demonstrate an important function of both in global metal homoeostasis in *C. metallidurans*.

## MATERIALS AND METHODS

### Bacterial strains and growth conditions.

Strains used for experiments were *C. metallidurans* plasmid-free derivative strain AE104 (13) and further derivatives of this strain as listed in Table 6. Tris-buffered mineral salts medium (13) containing 2 g ⋅ liter^−1^ sodium gluconate (TMM) was used to cultivate these strains aerobically with shaking at 30°C as previously published (21). The metal content of *C. metallidurans* cells was determined by inductively coupled plasma mass spectrometry (ICP-MS) using ESI sampler SC-2 (Elemental Scientific, Inc., Omaha, NE) and an X-Series II ICP-MS instrument (Thermo, Fisher Scientific, Bremen, Germany) as previously described in detail (10).

**TABLE 6:** Bacterial strains used^a^

Bacterial strain	Genotype	Reference
*Cupriavidus metallidurans*		
AE104	Plasmid-free parent strain	13
Δ*zupT* mutant	Δ*zupT*	2
Δ5	Δ*zupT ΔpitA ΔcorA*_1_ *ΔcorA*_2_ *ΔcorA*_3_	7
Δ7	Δ*zupT ΔpitA ΔcorA*_1_ *ΔcorA*_2_ *ΔcorA*_3_ *ΔzntB ΔhoxN*	7
Δ7A	Δ7 Δ*mgtA*::*kan*	This study
Δ8	Δ7 Δ*mgtB*	This study
Δ9	Δ8 *mgtA*::*kan* or Δ7 Δ*mgtB mgtA*::*kan*	This study
*Escherichia coli* S17/1	Conjugator strain	24

a Further derivatives such as Δ7 *mgtA*::*kan* or *lacZ* fusion strains are not listed. Construction of these strains is described in the supplemental material.

### Genetic techniques.

Standard molecular genetic techniques were used (22–24) as previously described (21). All primer pairs used are listed in Table S3 in the supplemental material. Deletion mutants within the same genome without interferences and secondary recombination events (25, 26) were also constructed as published previously (21). The correct deletions of the respective transporter genes were verified by Southern DNA-DNA hybridization. Dose-response growth curves and β-galactosidase assays in 96-well plates were conducted in TMM as published previously (21). The *lacZ* reporter gene was inserted within several target genes to construct reporter operon fusions as published previously (21).

10.1128/mSystems.00004-16.3 Table S3  Primers and plasmids used. Download Table S3, PDF file, 0.1 MB. Copyright © 2016 Große et al. 2016 Große et al. This content is distributed under the terms of the Creative Commons Attribution 4.0 International license.

### Transmission electron microscopy.

A preculture was incubated at 30°C, 250 rpm, for 30 h and then diluted 1:20 in fresh medium and incubated for 24 h at 30°C and 250 rpm. Cells were harvested by centrifugation at 5,000 rpm for 10 min and suspended in TMM. After incubation for 72 h at 30°C on a shaking incubator at 100 rpm, the cells were fixed directly with 3% glutaraldehyde (Sigma, Taufkirchen, Germany) in 0.1 M sodium cacodylate buffer (SCB) for 3 h, centrifuged at 5,000 rpm for 5 min, and taken up in 4% agar-SCB, followed by one washing step with SCB overnight at 4°C and three washing steps with the same buffer for 5 min. After postfixation with osmium tetroxide for 1 h, samples were dehydrated in a series of steps in ethanol (10%, 30%, and 50%). Then, cells were treated with 1% uranyl acetate-70% ethanol for 1 h and further dehydrated with a series of transfers from 70% to 90% to 100% ethanol. Thereafter, the samples were infiltrated with epoxy resin according to the method of Spurr (27) and polymerized at 70°C. The ultrathin sections (80 nm) were observed with an EM900 transmission electron microscope (Carl Zeiss SMT, Oberkochen, Germany) operating at 80 kV. The images were recorded using a Variospeed SSCCD SM-1k-120 camera (TRS, Moorenweis, Germany).

### Microarrays of *C. metallidurans*.

*C. metallidurans* AE104, Δ*zupT*, and Δ7 strains were treated with 10 µM zinc chloride or 50 µM EDTA in TMM. Each set of conditions was performed in triplicate, including three independent bacterial cultures for AE104 and Δ7 and two for the Δ*zupT* strain. RNA was isolated as described previously (21), quality checked, and provided to IMGM Laboratories GmbH (Martinsried, Germany) for hybridization with a *C. metallidurans* Agilent custom GE microarray (8 by 15K) (Agilent Technologies, Waldbronn, Germany) with a one-color (Cy3)-based protocol. Signals were detected using the Agilent DNA microarray scanner. Software tool Feature Extraction 10.7.3.1 was used for raw data extraction.

In the algorithm used, (i) the mean intensity of the pixels of the surrounding area was subtracted from the mean density of the pixels of the spots to give the signal strength. Its deviation was half of the sum of the two intensity deviations. The distance (*D*) value was the distance between spot and background pixel intensities divided by the sum of the deviations. *D* was a more useful value than Student’s *t* test because nontouching deviation bars of two values (*D* > 1) at three repeats always mean at least a significant (>95%) difference. Signals were further processed if *D* was >1. Following that, (ii) the mean values of the three biological repeats were calculated plus their deviation and the smallest *D* value (D_min). Third, (iii) the values from different spots, positions, or oligonucleotides assigned to the same gene were taken to calculate a gene-specific mean value and minimum *D* value. Finally, (iv) the respective spot signals coming from various growth conditions were compared (*Q* values). The *Q* values were the ratios of the mean signals from the various strains and conditions compared. *Q*(EDTA) of Δ7 and Δ*zupT* strains, for instance, was obtained by dividing the mean signal from EDTA-grown Δ7 cells by the mean signal from EDTA-grown Δ*zupT* cells (see Table S1 in the supplemental material). The cutoff value was a 2-fold upregulation (*Q* ≥ 2; see green letters in Table S1) or downregulation (*Q* ≤ 0.5; see red letters in Table S1). Moreover, the *D* values of these mean values were calculated according to the formula *D* = absolute(mean-1 − mean-2)/(deviation-1 + deviation-2). Nonsignificant *Q* values were accompanied by *D* < 1 and are given in italics in Table S1 in the supplemental material.

### Microarray data accession numbers.

The microarray data were deposited in the GEO database at Gene Expression Omnibus (http://www.ncbi.nlm.nih.gov/geo/) under accession numbers GSE64196 and GSE71011.
