# Supplementary material for: Characterization of the Δ7 Mutant of Cupriavidus metallidurans with Deletions of Seven Secondary Metal Uptake Systems
Source: mSystems. 2016 Feb 25;1(1):e00004-16. doi: 10.1128/mSystems.00004-16 (PMC5069749; doi:10.1128/mSystems.00004-16)
Supplement: Table S1 [file sys001162005st1.pdf]

Supplementary Table S1. Comparison of the transcriptomes of the strains AE104, Δ*zupT* and Δ7.

For the gene array experiment, strain Δ7, ΔzupT and AE104 parent were cultivated in TMM including 50 µM EDTA or 10 µM Zn(II)
RNA was isolated, reversely transcribed and used in a gene array experiment. 3 biological replicates for AE104 and Δ7, two for ΔzupT.
Ratios Q of the mean valies and D values given. Differences were counted as significant if (Q > 2 or Q < 0.5)AND(D > 1).
Up-regulated Q values in green, down-regulated in red, bold of D > 1, *italic if not.*
Protein numbers per cell from Herzberg et al., (2014) Metallomics 6, 2157-2165. NeF, never found; NF, not found in the respective strain; NQ, could not be quantified

Comparisons: Ratios Q of the mean gene array signal strain 1/strain 2, 50 µM EDTA or 10 µM Zn(II).

D values of the Q values in the respective left row: D = ABS(Q1-Q2)/(Stabw1+Stabw2)

Replicor	OpRegion	Q(EDTA)	Q(Zn)	Q(EDTA)	Q(Zn)	Q(AE104)	Gene	Name	Proteins per cell		Description
Rep	OpRegion	Δ7/ΔzupT	Δ7/ΔzupT	Δ7/AE104	Δ7/AE104	EDTA/Zn			AE104	ΔzupT	
**I. Up-regulated in Q(EDTA:Δ7/ΔzupT)**											
**IA. Up in EDTA, Up in Zn(II)**											
Rep1	Op0373f	**3.46**	**2.59**	1.01	1.25	1.06	-	Rmet_1301	354	NF/NF	Q1LNW1 Integrase, catalytic region
Rep1	Op0708r	**2.39**	**4.42**	**2.49**	**4.46**	1.13	-	Rmet_2556	NeF	NeF	Q1LK93 Integrase, catalytic region
Rep1	Op0734r	**2.65**	**2.40**	0.93	0.74	1.16	*zupT*	Rmet_2621	NeF	NeF	Q1LK28 Zinc/iron permease
Rep1	Op0810r	**2.20**	**2.91**	1.62	**2.49**	0.69	-	Rmet_2882	NeF	NeF	Q1LJC1 Putative uncharacterized protein
Rep1	Op0811f	**2.07**	**2.28**	1.40	1.69	0.72	-	Rmet_2883	NeF	NeF	Q1LJC0 RNA-directed DNA polymerase
Rep1	Op0860r	**3.10**	**6.72**	**2.93**	**7.44**	1.10	*mtgA*	Rmet_3053	34	NF/NF	Q1LIV1 Monofunctional biosynthetic peptidoglycan transglycosylase
**Environmental Information Processing**			**Membrane Transport**			**ABC transporters [PATH:ko02010]**					
Rep1	Op0895f	**2.72**	**6.47**	**2.23**	**5.39**	0.96	-	Rmet_3185	NeF	NeF	Q1LIG9 Binding-protein-dependent transport systems inner membrane component
Rep1	Op0895f	**4.28**	**7.24**	**3.25**	**5.58**	0.88	-	Rmet_3186	283	16	Q1LIG8 ABC transporter-related protein
Rep1	Op0896r	**2.18**	**2.31**	1.60	1.44	0.78	-	Rmet_3187	43	NF/NF	Q1LIG7 Putative uncharacterized protein
**Environmental Information Processing**			**Cell Motility**			**Flagellar assembly [PATH:ko02040]**					
Rep2	Op1026f	**2.19**	**3.02**	1.79	**2.51**	**2.01**	*flhD2*	Rmet_3685	17	NF/NF	Q1LH19 Flagellar transcriptional activator
Rep2	Op1026f	**2.07**	**2.32**	1.92	1.97	**2.16**	*flhC2*	Rmet_3686	NeF	NeF	Q1LH18 Flagellar transcriptional activator FlhC
Rep2	Op1026f	**2.27**	**2.61**	1.90	**2.25**	**2.36**	*motA*	Rmet_3687	NeF	NeF	Q1LH17 Chemotaxis MotA protein
Rep2	Op1026f	**2.43**	**2.62**	1.42	1.38	1.44	*flhG*	Rmet_3701	NeF	NeF	Q1LH03 Flagellar biosynthesis protein FlhG
Rep2	Op1042f	**2.23**	**2.02**	1.18	1.35	1.93	*flgF*	Rmet_3738	NF/NF	101	Q1LGW6 Flagellar basal-body rod FlgF
Rep2	Op1090f	**3.20**	**8.49**	**2.97**	**7.19**	0.87	-	Rmet_3902	1190	1384	Q1LGF6 Short-chain dehydrogenase/reductase SDR
Rep2	Op1162f	**2.02**	**3.61**	1.80	**3.44**	1.09	-	Rmet_4125	NF/NF	NF/NF	Q1LFT4 Putative uncharacterized protein
Rep2	Op1324f	**2.54**	**3.35**	0.79	0.95	1.34	-	Rmet_4603	123	3703	Q1LEG1 Transcriptional regulator, GntR family
Rep2	Op1324f	**2.26**	**2.66**	**0.46**	0.68	**2.10**	-	Rmet_4604	NeF	NeF	Q1LEG0 Putative uncharacterized protein
Rep2	Op1326f	**2.26**	**2.14**	1.26	1.22	1.41	*metY*	Rmet_4606	2698	174	Q1LEF8 O-acetylhomoserine/O-acetylserine sulfhydrylase
Rep2	Op1330f	**2.09**	**2.29**	1.00	1.00	0.98	-	Rmet_4616	NF/NF	154	Q1LEE8 Putative uncharacterized protein
Rep2	Op1411r	*2.20*	**2.47**	1.34	1.38	0.57	-	Rmet_4836	NeF	NeF	Q1LDS8 Diguanylate phosphodiesterase (EAL domain)
Rep2	Op1596f	*2.30*	**2.62**	1.36	1.35	0.56	-	Rmet_5365	NeF	NeF	Q1LCA2 Transcriptional regulator, LysR family
Rep2	Op1799r	**3.55**	**5.15**	**3.26**	**5.11**	1.08	-	Rmet_5924	63	NF/NF	Q1LAP3 SlyX
Rep2	Op1799r	**6.44**	**11.87**	**6.48**	**11.19**	1.06	-	Rmet_5925	396	572	Q1LAP2 3-demethylubiquinone-9 3-methyltransferase
**IB. Up in EDTA, down in Zn(II)**											
Rep1	Op0638r	*6.94*	*0.42*	*6.63*	*0.11*	*0.14*	-	Rmet_2228	NF/NF	NF/NF	Q1LL69 Putative uncharacterized protein
Rep1	Op0663f	*2.32*	*0.36*	1.92	1.91	1.02	-	Rmet_2345	NeF	NeF	Q8GQ55 Putative uncharacterized protein ORF C55
**IC. Up in EDTA, no change in Zn(II)**											
Rep1	Op0002r	*2.23*	1.07	1.92	*0.42*	0.50	-	Rmet_0008	11	NF/NF	Q1LSI2 Binding-protein-dependent transport systems inner membrane component
Rep1	Op0036r	*5.32*	0.83	*4.03*	*0.14*	*0.19*	*regA*	Rmet_0135	522	479	Q1LS55 Response regulator receiver domain protein (CheY-like)
Rep1	Op0066r	*2.97*	1.05	*2.96*	*0.40*	*0.34*	-	Rmet_0221	NeF	NeF	Q1LRW9 Putative uncharacterized protein

Rep1	Op0096r	*3.08*	0.93	*2.84*	*0.31*	*0.34*	-	Rmet_0343	184	224	Q1LRJ7 Transcriptional regulator, RpiR family
Rep1	Op0118r	*2.37*	1.02	*2.05*	*0.40*	*0.40*	-	Rmet_0455	NeF	NeF	Q1LR85 Cl-channel, voltage gated
Rep1	Op0140r	*2.46*	0.75	*2.19*	*0.45*	*0.47*	-	Rmet_0522	105	174	Q1LR18 Putative uncharacterized protein
Rep1	Op0162r	*2.45*	1.23	*2.27*	*0.45*	*0.43*	-	Rmet_0576	NeF	NeF	Q1LQW4 Fimbrial protein
Rep1	Op0172r	*2.32*	0.78	*2.18*	*0.38*	*0.47*	-	Rmet_0607	198	212	Q1LQT3 Hydroxymethylglutaryl-CoA lyase
Rep1	Op0179f	*2.62*	1.14	*2.47*	*0.44*	*0.47*	-	Rmet_0626	NF/NF	602	Q1LQR4 Rhs element Vgr protein
Rep1	Op0180r	*2.31*	1.19	1.67	*0.47*	0.60	-	Rmet_0650	NeF	NeF	Q1LQP0 Peptidase A24A, prepilin type IV
Rep1	Op0184r	*2.62*	0.88	*2.47*	*0.37*	*0.36*	-	Rmet_0664	38	NF/NF	Q1LQM6 Putative uncharacterized protein
Rep1	Op0211f	*2.07*	0.88	0.92	*0.45*	0.98	-	Rmet_0745	NeF	NeF	Q1LQE5 Putative uncharacterized protein
Rep1	Op0220r	*2.20*	1.15	1.96	*0.49*	0.51	*phnE*	Rmet_0773	NeF	NeF	Q1LQB7 Phosphonate uptake transporter
Rep1	Op0299f	*3.01*	1.00	*2.72*	*0.50*	*0.35*	*lolE*	Rmet_1040	576	47	Q1LPK0 Lipoprotein releasing system, transmembrane protein, LolC/E family
Rep1	Op0325f	*2.09*	1.17	1.65	0.72	*0.49*	-	Rmet_1139	NeF	NeF	Q1LPA1 Major facilitator superfamily MFS_1
Rep1	Op0362r	**2.07**	1.42	0.93	0.94	1.08	-	Rmet_1251	NQ/NQ	NF/NF	Q1LNY9 Putative uncharacterized protein
Rep1	Op0370r	*2.07*	1.05	1.90	*0.43*	*0.49*	*hoxG*	Rmet_1297	NF/NF	NF/NF	Q1LNU4 Nickel-dependent hydrogenase, large subunit
Rep1	Op0386r	*2.04*	0.96	1.98	*0.44*	*0.49*	-	Rmet_1386	NeF	NeF	Q1LNK7 Putative uncharacterized protein
Rep1	Op0397f	*2.44*	1.13	*2.04*	0.58	*0.44*	*dppB1*	Rmet_1409	35	NF/NF	Q1LNI4 Binding-protein-dependent transport systems inner membrane component
Rep1	Op0485f	*2.37*	1.07	*2.10*	*0.45*	*0.47*	*tauD*	Rmet_1717	15	NF/NF	Q1LMM8 Taurine catabolism dioxygenase TauD/TfdA
Rep1	Op0492r	*3.04*	1.06	*2.29*	*0.27*	*0.40*	-	Rmet_1734	NeF	NeF	Q1LML3 Putative uncharacterized protein
Rep1	Op0587f	*2.16*	1.12	1.83	*0.46*	0.53	-	Rmet_2006	NeF	NeF	Q1LLU1 Putative uncharacterized protein
Rep1	Op0663f	*2.07*	1.21	1.20	0.62	0.70	-	Rmet_2350	NeF	NeF	Q8GQ60 Putative uncharacterized protein ORF C50
Rep1	Op0664r	*2.20*	0.76	0.87	**0.34**	1.06	-	Rmet_2361	NeF	NeF	Q8GQ71 Putative uncharacterized protein ORF C39
Rep1	Op0688r	*2.00*	0.74	1.32	0.60	0.70	-	Rmet_2461	NeF	NeF	Q1LKI8 Colicin V production protein
Rep1	Op0696r	*2.34*	0.94	*2.11*	*0.42*	*0.46*	-	Rmet_2518	NeF	NeF	Q1LKD1 Chromate transporter
Rep1	Op0754r	*2.39*	1.07	*2.31*	*0.44*	*0.46*	-	Rmet_2661	NeF	NeF	Q1LJZ2 Putative uncharacterized protein
Rep1	Op0776r	*8.63*	1.07	*8.03*	0.83	0.81	-	Rmet_2759	NeF	NeF	Q1LJP4 Putative lipoprotein
Rep1	Op0780r	*2.94*	1.08	*2.40*	*0.35*	*0.47*	-	Rmet_2775	36	NF/NF	Q1LJM8 PepSY-associated TM helix
Rep1	Op0788r	*2.96*	0.75	*2.13*	*0.27*	*0.43*	-	Rmet_2802	60	NF/NF	Q1LJK1 Prolipoprotein diacylglyceryl transferase
Rep1	Op0848r	*3.89*	1.19	*3.49*	*0.25*	*0.28*	-	Rmet_3025	NeF	NeF	Q1LIX8 Putative uncharacterized protein
Rep1	Op0956r	*2.09*	1.01	1.94	0.74	0.83	-	Rmet_3439	NeF	NeF	Q1LHR5 GCN5-related N-acetyltransferase
Rep1	Op0959f	*4.42*	0.95	*4.72*	*0.21*	*0.22*	*dctA2*	Rmet_3444	NeF	NeF	Q1LHR0 Sodium:dicarboxylate symporter
**Cellular**	**Processes**			**Cell Motility**		**Bacterial chemotaxis [PATH:ko02030]**					
Rep2	Op1026f	*2.87*	1.22	*2.32*	*0.48*	*0.46*	-	Rmet_3679	177	NF/NF	Q1LH25 Diguanylate cyclase/phosphodiesterase
Rep2	Op1026f	**2.14**	1.56	1.12	1.07	1.88	*cheW*	Rmet_3681	92	NF/NF	Q1LH23 CheW protein
Rep2	Op1026f	**2.56**	1.77	1.41	1.12	1.77	-	Rmet_3684	NF/NF	207	Q1LH20 Putative uncharacterized protein
Rep2	Op1026f	**2.02**	1.78	1.49	1.38	**2.10**	*cheW*	Rmet_3690	93	NF/NF	Q1LH14 CheW protein
Rep2	Op1026f	**2.14**	1.73	1.49	1.20	1.65	*cheR*	Rmet_3691	NeF	NeF	Q1LH13 MCP methyltransferase, CheR-type
Rep2	Op1026f	**2.05**	1.68	1.53	1.44	1.83	*cheZ*	Rmet_3695	187	374	Q1LH09 Chemotaxis phosphatase, CheZ
Rep2	Op1026f	**2.93**	1.51	1.97	0.55	0.63	*fliA*	Rmet_3702	NeF	NeF	Q1LH02 RNA polymerase sigma factor
Rep2	Op1042f	**2.05**	1.84	1.02	1.40	**2.37**	*flgB*	Rmet_3734	145	NF/NF	Q1LGX0 Flagellar basal-body rod protein FlgB
Rep2	Op1042f	**2.08**	1.73	1.04	1.23	**2.19**	*flgC*	Rmet_3735	NeF	NeF	Q1LGW9 Flagellar basal-body rod protein FlgC
Rep2	Op1047r	*2.52*	1.04	*2.28*	*0.35*	*0.43*	*alkA*	Rmet_3766	NeF	NeF	Q1LGT8 Putative uncharacterized protein
Rep2	Op1053r	*2.86*	1.05	*2.61*	*0.34*	*0.38*	*lipA*	Rmet_3796	NeF	NeF	Q1LGR0 Alpha/beta hydrolase fold
Rep2	Op1057r	*2.16*	1.23	*2.07*	*0.46*	*0.46*	-	Rmet_3801	297	7	Q1LGQ5 Uncharacterized protein UPF0065
Rep2	Op1066f	*2.02*	1.37	1.93	*0.49*	*0.44*	-	Rmet_3841	NeF	NeF	Q1LGL7 Tannase and feruloyl esterase
Rep2	Op1082f	*2.22*	0.97	*2.19*	0.51	*0.46*	-	Rmet_3883	NeF	NeF	Q1LGH5 Putative uncharacterized protein
Rep2	Op1125r	*2.27*	0.89	1.99	*0.39*	*0.47*	-	Rmet_4007	NeF	NeF	Q1LG51 Putative uncharacterized protein
Rep2	Op1141r	*2.30*	1.22	1.96	*0.35*	*0.41*	-	Rmet_4057	NeF	NeF	Q1LG01 CcmE/CycJ protein
Rep2	Op1143r	*2.34*	1.20	*2.25*	0.64	0.63	-	Rmet_4070	36	NF/NF	Q1LFY8 Transcriptional regulator, LysR family
Rep2	Op1165r	*8.17*	1.00	*5.65*	*0.13*	*0.18*	*pdhA*	Rmet_4134	NeF	NeF	Q1LFS5 Dehydrogenase, E1 component
Rep2	Op1170f	*2.07*	1.15	1.54	0.51	0.59	-	Rmet_4157	72	NF/NF	Q1LFQ2 Putative lipoprotein
Rep2	Op1175r	*2.76*	1.16	1.86	*0.39*	0.56	-	Rmet_4173	NeF	NeF	Q1LFN6 Putative uncharacterized protein

Rep2	Op1204f	2.51	1.20	2.13	0.41	0.44	-	Rmet_4251	NeF	NeF	Q1LFF9 Pili assembly chaperone
Rep2	Op1257r	2.16	0.95	2.01	0.38	0.42	-	Rmet_4411	NeF	NeF	Q1LF00 CoA-binding
Rep2	Op1263r	4.83	1.01	3.84	0.19	0.22	-	Rmet_4437	NeF	NeF	Q1LEX4 Putative uncharacterized protein
Rep2	Op1271r	5.82	0.99	4.13	0.24	0.20	-	Rmet_4461	NeF	NeF	Q1LEV0 Putative uncharacterized protein
Rep2	Op1278f	2.04	1.30	1.84	0.52	0.51	-	Rmet_4483	14	NF/NF	Q1LES8 Phenylacetic acid degradation-related protein
Rep2	Op1314f	2.05	1.16	0.76	1.01	1.51	-	Rmet_4565	91	50	Q1LEJ9 TonB-dependent receptor
**Environmental Information Processing**				**Membrane Transport**		**Pores ion channels**					
Rep2	Op1330f	**3.63**	1.40	1.25	0.65	**8.91**	-	Rmet_4617	NF/NF	NF/NF	Q1LEE7 TonB-dependent siderophore receptor
Rep2	Op1331r	**3.50**	1.61	1.15	0.75	**12.11**	-	Rmet_4618	NeF	NeF	Q1LEE6 Putative uncharacterized protein
Rep2	Op1419r	3.17	0.95	3.14	0.32	0.31	-	Rmet_4863	NeF	NeF	Q1LDQ1 Putative uncharacterized protein
Rep2	Op1439r	3.16	0.96	3.04	0.27	0.30	-	Rmet_4913	NeF	NeF	Q1LDK1 ABC-type transport system protein
Rep2	Op1540f	2.14	1.20	1.62	0.54	0.60	*hipO*	Rmet_5222	NeF	NeF	Q1LCP5 Peptidase M20D, amidohydrolase
Rep2	Op1544f	**2.11**	1.55	1.17	1.16	1.47	-	Rmet_5236	NeF	NeF	Q1LCN1 DegT/DnrJ/EryC1/StrS aminotransferase
Rep2	Op1550f	**2.57**	1.39	1.03	1.02	**2.57**	*fliC2*	Rmet_5252	2179	76	Q1LCL5 Flagellin-like protein
Rep2	Op1552f	2.57	0.80	1.92	0.38	0.56	*fliK*	Rmet_5266	NeF	NeF	Q1LCK1 Flagellar hook-length control protein
Rep2	Op1592f	2.15	1.16	1.77	0.73	0.65	-	Rmet_5359	NeF	NeF	Q1LCA8 Uncharacterized protein UPF0065
Rep2	Op1619r	2.94	0.80	3.03	0.39	0.40	-	Rmet_5445	59	NF/NF	Q1LC22 Carbohydrate kinase, FGGY
Rep2	Op1706f	10.62	0.70	8.67	0.13	0.14	-	Rmet_5706	NeF	NeF	Q1LBB1 Putative uncharacterized protein
Rep2	Op1763r	2.16	0.97	2.00	0.51	0.56	*pstS1*	Rmet_5829	NeF	NeF	Q1LAY8 Periplasmic phosphate binding protein
Rep2	Op1779r	110.09	1.19	95.83	1.05	1.07	-	Rmet_5879	NeF	NeF	Q1LAT8 Transcriptional regulator, LysR family
Rep2	Op1783r	6.39	1.07	5.43	0.20	0.20	-	Rmet_5886	147	NF/NF	Q1LAT1 OmpW

II.Down-regulated in Q(EDTA:Δ7/ΔzupT)

IIA. Down-regulated in Q(EDTA:Δ7/ΔzupT) AND Down-regulated in Q(Zn:Δ7/ΔzupT) AND (Down-regulated or not-regulated) in Q(Δ7/AE104)

Rep1	Op0427f	**0.02**	**0.02**	**0.14**	**0.24**	1.65	*hoxN*	Rmet_1533	NeF	NeF	Q1LN60 High-affinity nickel-transporter
Rep1	Op0935f	**0.37**	**0.42**	**0.06**	**0.09**	**2.13**	-	Rmet_3359	NeF	NeF	Q1LHZ5 UBA/THIF-type NAD/FAD binding fold
Rep1	Op0974r	**0.41**	**0.42**	0.63	0.58	**2.07**	*livF5*	Rmet_3519	556	344	Q1LHI5 ABC transporter-related protein

IIB. Down-regulated in Q(EDTA:Δ7/ΔzupT) AND not regulated in Q(Zn:Δ7/ΔzupT) AND Up-regulated in Q(EDTA:Δ7/AE104)

cobW1 gene cluster

Rep1	Op0317f	**0.30**	1.23	**8.01**	0.87	1.85	-	Rmet_1098	NeF	NeF	Q1LPE2 Cobalamin synthesis protein, P47K
Rep1	Op0317f	**0.28**	1.76	**9.55**	0.92	1.84	-	Rmet_1099	NeF	NeF	Q1LPE1 UPF0343 protein Rmet_1099
Rep1	Op0317f	**0.27**	1.25	**8.65**	0.87	**2.26**	*cysS*	Rmet_1100	NeF	NeF	Q1LPE0 Cysteinyl-tRNA synthetase
Rep1	Op0317f	**0.27**	1.24	**5.79**	0.92	1.57	-	Rmet_1101	NeF	NeF	Q1LPD9 6-pyruvoyl-tetrahydropterin synthase-like protein
Rep1	Op0317f	**0.27**	1.17	**6.28**	0.71	1.15	-	Rmet_1102	NF/NF	NF/NF	Q1LPD8 Carbonic anhydrases/acetyltransferase isoleucine patch superfamily
Rep1	Op0317f	**0.32**	1.24	**6.50**	0.91	1.85	*allB*	Rmet_1103	NeF	NeF	Q1LPD7 Dihydroorotase

IIC. Down-regulated in Q(EDTA:Δ7/ΔzupT) AND not regulated in Q(Zn:Δ7/ΔzupT) AND NOT Up-regulated in Q(EDTA:Δ7/AE104)

Rep1	Op0178r	-3.64	1.28	-3.11	0.97	0.99	-	Rmet_0625	NeF	NeF	Q1LQR5 Uncharacterised conserved protein UCP028301
Rep1	Op0232r	-1.47	1.08	0.83	0.88	0.96	*ggt*	Rmet_0822	897	901	Q1LQ68 Gamma-glutamyltransferase 2. Threonine peptidase. MEROPS family T03
Rep1	Op0318r	**0.35**	1.10	1.74	0.69	1.89	-	Rmet_1104	NeF	NeF	Q1LPD6 TonB-dependent siderophore receptor
Rep1	Op0370r	0.33	1.35	0.89	0.97	1.04	-	Rmet_1290	NF/NF	NF/NF	Q1LNV1 HupH hydrogenase expression protein
Rep1	Op0701f	0.39	1.30	1.01	1.02	1.00	-	Rmet_2538	33	NF/NF	Q1LKB1 Porin, Gram-negative type
Rep1	Op0782r	-1.99	1.06	-2.02	1.01	0.82	-	Rmet_2777	36	NF/NF	Q1LJM6 Cobyrinic acid a,c-diamide synthase
Rep1	Op0886r	-5.20	0.78	-5.30	0.96	1.06	*paaF*	Rmet_3162	1061	1332	Q1LIJ2 Short chain enoyl-CoA hydratase
Rep1	Op0935f	**0.40**	0.64	**0.41**	0.71	1.05	-	Rmet_3356	75	NF/NF	Q1LHZ8 ATP-binding region, ATPase-like protein
Rep1	Op0962r	0.46	0.89	1.04	0.82	0.94	-	Rmet_3460	NeF	NeF	Q1LHP4 Putative uncharacterized protein
Rep2	Op1132f	0.41	1.11	0.83	1.03	0.82	-	Rmet_4025	NeF	NeF	Q1LG33 Major facilitator superfamily MFS_1
Rep2	Op1248f	-0.98	1.09	-1.04	0.94	0.82	-	Rmet_4387	255	439	Q1LF24 Tryptophan halogenase
Rep2	Op1395r	0.48	1.17	0.94	0.97	0.95	-	Rmet_4790	NeF	NeF	Q1LDX4 RND efflux system, outer membrane lipoprotein, NodT

III. Not regulated in Q(EDTA:Δ7/ΔzupT) AND down-regulated in Q(Zn:Δ7/ΔzupT)

IIIA. Genetic Information Processing

IIIA1. Translation

IIIA1a. Ribosome

Rep1	Op0576r	1.12	**0.27**	0.88	0.50	0.53	*rplI*	Rmet_1976	8849	9065	Q1LLX1 50S ribosomal protein L9
Rep1	Op0576r	1.06	**0.27**	0.91	**0.50**	0.53	*rpsR*	Rmet_1977	5167	5626	Q1LLX0 30S ribosomal protein S18
Rep1	Op0576r	1.12	**0.32**	1.02	0.50	0.52	*priB*	Rmet_1978	56	53	Q1LLW9 Restart primosome assembly protein PriB
Rep1	Op0576r	0.79	**0.43**	0.80	0.68	0.68	*rpsF*	Rmet_1979	4861	5366	Q1LLW8 30S ribosomal protein S6
Rep1	Op0926r	1.14	*0.48*	1.01	0.72	**0.50**	*rplQ*	Rmet_3290	5888	6325	Q1LI64 50S ribosomal protein L17
Rep1	Op0926r	0.96	**0.41**	0.87	0.75	0.67	*rpsD*	Rmet_3292	7174	7959	Q1LI62 30S ribosomal protein S4
Rep1	Op0926r	1.18	**0.49**	0.95	0.66	0.73	*rpsK*	Rmet_3293	5353	5869	Q1LI61 30S ribosomal protein S11
Rep1	Op0926r	0.99	**0.45**	0.85	0.67	0.76	*rpsM*	Rmet_3294	4610	4997	Q1LI60 30S ribosomal protein S13
Rep1	Op0926r	0.96	**0.44**	0.85	0.64	0.76	*rpmJ*	Rmet_3295	NeF	NeF	Q1LI59 50S ribosomal protein L36
Rep1	Op0926r	0.96	**0.40**	0.73	0.55	0.99	*infA*	Rmet_3296	139	138	Q1LI58 Translation initiation factor IF-1 2
Rep1	Op0926r	1.12	**0.37**	0.82	0.51	0.70	*rplO*	Rmet_3298	6201	6664	Q1LI56 50S ribosomal protein L15
Rep1	Op0926r	1.08	**0.43**	0.86	0.56	0.66	*rpmD*	Rmet_3299	1634	3543	Q1LI55 50S ribosomal protein L30
Rep1	Op0926r	0.97	**0.44**	0.79	0.55	0.82	*rpsE*	Rmet_3300	5381	6434	Q1LI54 30S ribosomal protein S5
Rep1	Op0926r	1.07	**0.36**	0.77	0.51	0.65	*rplR*	Rmet_3301	4727	5155	Q1LI53 50S ribosomal protein L18
Rep1	Op0926r	1.47	*0.39*	0.82	**0.36**	0.52	*rplF*	Rmet_3302	7732	7720	Q1LI52 50S ribosomal protein L6
Rep1	Op0926r	1.20	**0.36**	0.79	**0.48**	0.63	*rpsH*	Rmet_3303	5099	5478	Q1LI51 30S ribosomal protein S8
Rep1	Op0926r	1.03	**0.28**	0.72	0.55	0.91	*rpsN*	Rmet_3304	567	1422	Q1LI50 SSU ribosomal protein S14P
Rep1	Op0926r	0.98	**0.27**	0.65	0.62	1.18	*rplE*	Rmet_3305	5648	5794	Q1LI49 50S ribosomal protein L5
Rep1	Op0926r	1.24	**0.38**	0.52	0.54	0.87	*rplX*	Rmet_3306	5157	5464	Q1LI48 50S ribosomal protein L24
Rep1	Op0926r	1.13	**0.39**	**0.43**	**0.48**	0.98	*rplN*	Rmet_3307	4613	4259	Q1LI47 50S ribosomal protein L14
Rep1	Op0926r	1.10	**0.38**	1.03	0.53	0.50	*rpsQ*	Rmet_3308	3251	4144	Q1LI46 30S ribosomal protein S17
Rep1	Op0926r	1.10	**0.32**	0.89	**0.49**	0.72	*rpmC*	Rmet_3309	6327	6645	Q1LI45 50S ribosomal protein L29
Rep1	Op0926r	1.02	**0.31**	0.92	0.51	0.61	*rplP*	Rmet_3310	7207	7507	Q1LI44 50S ribosomal protein L16
Rep1	Op0926r	0.93	**0.33**	0.86	0.71	0.92	*rpsC*	Rmet_3311	5821	5414	Q1LI43 30S ribosomal protein S3
Rep1	Op0926r	1.14	*0.46*	1.01	0.60	0.55	*rplV*	Rmet_3312	5052	5381	Q1LI42 50S ribosomal protein L22
Rep1	Op0926r	1.12	*0.44*	0.92	0.53	0.56	*rpsS*	Rmet_3313	3671	4605	Q1LI41 30S ribosomal protein S19
Rep1	Op0926r	1.17	**0.29**	0.87	0.55	0.89	*rplB*	Rmet_3314	8352	8668	Q1LI40 50S ribosomal protein L2
Rep1	Op0926r	1.15	**0.36**	0.89	0.66	0.63	*rplW*	Rmet_3315	7955	8287	B3R7S1 50S ribosomal subunit protein L23
Rep1	Op0926r	1.11	**0.32**	0.91	0.66	0.74	*rplD*	Rmet_3316	7471	7656	Q1LI38 50S ribosomal protein L4
Rep1	Op0926r	1.13	**0.41**	0.78	0.66	0.67	*rplC*	Rmet_3317	7040	7731	Q1LI37 50S ribosomal protein L3
Rep1	Op0928r	1.03	**0.42**	0.95	0.72	0.75	*rpsJ*	Rmet_3323	5007	5588	B3R7S9 30S ribosomal subunit protein S10
Rep1	Op0928r	1.16	**0.32**	0.75	0.63	0.86	-	Rmet_3324	14226	16250	Q1LI13 Elongation factor Tu
Rep1	Op0928r	1.03	**0.38**	0.99	0.61	0.64	-	Rmet_3325	7986	8299	Q1LI29 Elongation factor G 1
Rep1	Op0928r	1.14	**0.35**	0.98	0.62	0.55	*rpsG*	Rmet_3326	7924	9112	Q1LI28 30S ribosomal protein S7
Rep1	Op0928r	1.21	**0.33**	0.97	0.53	0.59	*rpsL*	Rmet_3327	4440	4693	Q1LI27 30S ribosomal protein S12
Rep1	Op0930r	1.02	**0.47**	0.73	0.69	0.89	*rplJ*	Rmet_3336	5462	5628	Q1LI18 50S ribosomal protein L10
Rep1	Op0930r	1.27	**0.41**	0.82	0.56	0.77	*rplA*	Rmet_3337	9005	9349	Q1LI17 50S ribosomal protein L1
Rep1	Op0930r	1.17	**0.36**	0.68	0.63	0.91	*rplK*	Rmet_3338	4798	4974	Q1LI16 50S ribosomal protein L11
Rep1	Op0930r	1.07	**0.42**	0.60	0.64	1.12	*nusG*	Rmet_3339	3359	4005	Q1LI15 Transcription antitermination protein nusG
Rep1	Op0874r	1.17	**0.41**	0.68	0.71	0.92	*rpmA*	Rmet_3105	2121	2882	Q1LIP9 50S ribosomal protein L27
Rep1	Op0874r	1.04	**0.45**	0.72	0.79	1.01	*rplU*	Rmet_3106	5361	5288	Q1LIP8 50S ribosomal protein L21
Rep1	Op0594r	1.00	**0.45**	0.88	0.68	0.75	*infB*	Rmet_2031	2797	2864	Q1LLR6 Translation initiation factor IF-2
Rep1	Op0594r	0.93	**0.40**	0.60	0.64	1.08	-	Rmet_2033	1037	1276	Q1LLR4 UPF0090 protein Rmet_2033
Rep1	Op0594r	0.86	**0.49**	0.78	0.67	1.02	-	Rmet_2035	597	512	Q1LLR2 Condensin subunit ScpB
Rep1	Op0680r	1.10	**0.42**	0.76	0.68	0.78	*rpmF*	Rmet_2432	3633	4851	Q1LKL7 50S ribosomal protein L32

Rep1	Op0084r	1.17	**0.34**	0.92	0.57	0.68	*rplY*	Rmet_0288	7358	8076	Q1LRQ2	50S ribosomal protein L25
Rep1	Op0084r	1.17	**0.34**	0.92	0.57	0.68	*rplY*	Rmet_0288	7358	8076	Q1LRQ2	50S ribosomal protein L25
**IIIA1b. Other translation proteins**												
Rep1	Op0016r	1.08	**0.49**	1.03	0.70	0.81	*gatB*	Rmet_0046	1051	1250	Q1LSE4	Aspartyl/glutamyl-tRNA(Asn/Gln) amidotransferase subunit B
**IIIA2 Folding, Sorting and Degradation**												
Rep1	Op0926r	1.16	**0.40**	0.84	0.51	0.64	*secY*	Rmet_3297	882	376	Q1LI57	Preprotein translocase secY subunit
Rep1	Op0544r	0.82	**0.45**	0.85	0.79	1.14	-	Rmet_1883	1666	1708	Q1LM64	ATP-dependent protease La
Rep1	Op0544r	1.23	**0.46**	0.97	0.71	0.54	*tig*	Rmet_1886	5656	6401	Q1LM61	Trigger factor
Rep1	Op0918r	0.79	**0.38**	1.04	0.79	0.63	-	Rmet_3234	792	350	Q1LIC0	Porin, Gram-negative type
Rep1	Op0918r	0.90	**0.37**	0.88	0.73	0.97	*tatA*	Rmet_3237	1578	883	Q1LIB7	Sec-independent protein translocase protein tatA/E homolog
Rep1	Op0918r	1.09	**0.46**	0.98	0.66	0.72	-	Rmet_3251	365	459	Q1LIA3	BolA-like protein
**IIIA3. Transcription**												
Rep1	Op0594r	1.00	**0.42**	0.76	0.69	0.82	*nusA*	Rmet_2032	2898	3188	Q1LLR5	NusA antitermination factor
Rep1	Op0930r	0.90	**0.40**	1.01	0.68	0.81	*rpoC*	Rmet_3333	4709	4801	Q1LI21	DNA-directed RNA polymerase subunit beta'
Rep1	Op0930r	0.75	**0.47**	0.79	0.67	0.97	*rpoB*	Rmet_3334	4908	5411	Q1LI20	DNA-directed RNA polymerase subunit beta
Rep1	Op0926r	1.08	**0.47**	0.97	0.71	0.63	*rpoA*	Rmet_3291	6895	8414	Q1LI63	DNA-directed RNA polymerase subunit alpha
Rep1	Op0730r	1.11	**0.46**	0.93	0.71	0.75	*rpoD1*	Rmet_2606	1927	1521	Q1LK43	RNA polymerase sigma factor
**IIIA4. Replication and Repair**												
Rep2	Op1377r	0.86	**0.40**	1.26	1.09	1.24	-	Rmet_4749	6291	7312	Q46TY9	HU family DNA-binding protein
Rep2	Op1628f	0.97	*0.00*	0.90	0.99	1.14	*dam*	Rmet_5483	NeF	NeF	Q1LBY4	DNA adenine methylase Dam
**IIIB. Metabolism**												
**IIIB1. Energy Metabolism**												
**IIIB1a. Oxidative phosphorylation [PATH:ko00190]**												
Rep1	Op0950r	1.13	**0.36**	1.09	0.65	0.60	-	Rmet_3421	695	585	Q1LHT3	Conserved hypothetical signal peptide protein
Rep1	Op0950r	0.98	**0.34**	0.94	0.80	0.70	-	Rmet_3422	1258	828	Q1LHT2	Sulfur compound chelating protein SoxZ
Rep1	Op0950r	0.89	**0.38**	0.88	0.82	0.57	-	Rmet_3423	1496	966	Q1LHT1	Twin-arginine translocation pathway signal
Rep1	Op0950r	1.21	**0.40**	1.10	0.76	**0.35**	-	Rmet_3424	4227	2434	Q1LHT0	Cytochrome c, class I
Rep1	Op0970r	0.66	**0.44**	0.76	0.73	0.99	-	Rmet_3487	416	348	Q1LHL7	Tetratricopeptide TPR_4
Rep1	Op0970r	0.98	**0.37**	0.86	0.56	0.73	*atpD*	Rmet_3494	7664	6955	Q1LHL0	ATP synthase subunit beta
Rep1	Op0970r	0.96	**0.31**	0.96	0.56	0.61	*atpG*	Rmet_3495	2406	2347	Q1LHK9	ATP synthase gamma chain
Rep1	Op0970r	1.01	**0.35**	0.96	0.67	0.62	*atpA*	Rmet_3496	5312	4653	Q1LHK8	ATP synthase subunit alpha
Rep1	Op0970r	0.79	**0.36**	0.85	0.68	0.73	*atpH*	Rmet_3497	1892	1624	Q1LHK7	ATP synthase F1, delta subunit
Rep1	Op0970r	1.01	**0.36**	0.82	0.64	0.75	*atpF*	Rmet_3498	1934	1021	Q1LHK6	ATP synthase B chain
Rep1	Op0970r	0.89	**0.26**	0.74	0.62	0.92	*atpE*	Rmet_3499	601	104	Q1LHK5	ATP synthase F0, C subunit
Rep1	Op0970r	0.91	**0.45**	0.82	0.66	0.87	*atpB*	Rmet_3500	778	274	Q1LHK4	ATP synthase A chain
Rep1	Op0077f	0.65	**0.38**	0.92	0.60	1.23	*ctaB*	Rmet_0270	NeF	NeF	Q1LRS0	Protoheme IX farnesyltransferase
Rep1	Op0914r	0.85	**0.41**	0.85	0.91	1.00	*petA*	Rmet_3230	1409	1046	Q1LIC4	Ubiquinol-cytochrome c reductase, iron-sulfur subunit
**IIIIB1b. Sulfur metabolism [PATH:ko00920]**												
Rep1	Op0792r	1.08	**0.48**	1.11	0.76	0.70	*cysN*	Rmet_2812	1622	1395	Q1LJJ1	Sulfate adenylyltransferase subunit 1
Rep1	Op0792r	0.78	**0.44**	0.85	0.68	0.86	*cysD*	Rmet_2813	1292	1269	Q1LJJ0	Sulfate adenylyltransferase subunit 2
Rep1	Op0792r	0.99	**0.46**	1.07	0.83	0.71	-	Rmet_2815	1546	1827	Q1LJI8	Uncharacterised conserved protein UCP030820
**IIIIB1c. Nitrogen metabolism [PATH:ko00910]**												
Rep1	Op0438r	0.73	**0.32**	0.85	0.62	1.16	*ansB*	Rmet_1583	3456	3637	Q1LN10	Asparaginase
Rep1	Op0920r	0.96	**0.48**	0.99	0.88	0.71	*gltD*	Rmet_3262	4014	4755	Q1LI92	Glutamate synthase (NADH) small subunit
**IIIB2. Carbohydrate Metabolism**												
**IIIB2a. Citrate cycle (TCA cycle) [PATH:ko00020]**												
Rep1	Op0596r	1.33	**0.44**	1.28	*0.39*	0.72	*sucB*	Rmet_2049	4472	4881	Q1LLP8	2-oxoglutarate dehydrogenase E2 component
Rep1	Op0596r	0.93	**0.38**	0.85	0.79	0.88	*kgd*	Rmet_2050	4358	4654	Q1LLP7	2-oxoglutarate dehydrogenase E1 component
Rep1	Op0596r	0.94	**0.43**	0.91	0.82	0.80	*kdg*	Rmet_2050	4358	4654	Q1LLP7	2-oxoglutarate dehydrogenase E1 component
Rep1	Op0596r	0.97	**0.44**	0.95	0.79	0.77	*kgd0*	Rmet_2050	4358	4654	Q1LLP7	2-oxoglutarate dehydrogenase E1 component
Rep2	Op1041r	0.75	**0.33**	1.06	0.79	1.10	*icdA*	Rmet_3729	7557	9298	Q1LGX5	Isocitrate dehydrogenase, NADP-dependent

	**IIIB2b. Citrate cycle and amino acid biosynthesis**											
Rep1	Op0688r	1.16	**0.41**	1.02	0.67	0.87	*asd*	Rmet_2471	4796	5428	Q1LKH8	Aspartate semialdehyde dehydrogenase
Rep1	Op0688r	0.90	**0.43**	0.81	0.76	0.62	*leuB*	Rmet_2472	2330	3043	Q1LKH7	3-isopropylmalate dehydrogenase
Rep1	Op0688r	1.05	**0.48**	0.96	0.78	0.61	*leuC*	Rmet_2475	1783	2038	Q1LKH4	3-isopropylmalate dehydratase, large subunit
Rep1	Op0688r	0.76	**0.38**	0.91	0.62	0.72	*livG*	Rmet_2477	772	261	Q1LKH2	ABC transporter-related protein
Rep1	Op0688r	0.66	**0.26**	1.07	0.82	0.75	*livK1*	Rmet_2480	11003	10023	Q1LKG9	Extracellular ligand-binding receptor
Rep1	Op0688r	1.08	**0.37**	1.03	0.69	0.51	*gltA*	Rmet_2481	8951	9563	Q1LKG8	Citrate synthase
Rep1	Op0688r	1.17	**0.35**	1.00	0.63	**0.47**	-	Rmet_2482	309	355	Q1LKG7	Putative uncharacterized protein
Rep1	Op0688r	0.96	**0.43**	0.79	0.73	0.58	*sdhB*	Rmet_2483	4739	3592	Q1LKG6	Succinate dehydrogenase subunit B
Rep1	Op0688r	1.11	**0.47**	0.74	0.63	**0.38**	*sdhA*	Rmet_2484	6561	5524	Q1LKG5	Succinate dehydrogenase subunit A
Rep1	Op0688r	1.33	**0.49**	0.72	0.62	**0.38**	*sdhD*	Rmet_2485	326	NF/NF	Q1LKG4	Succinate dehydrogenase subunit D
Rep1	Op0280r	0.63	**0.28**	0.96	1.09	1.23	*aroG*	Rmet_0989	1309	2399	Q1LPQ1	3-deoxy-D-arabinoheptulosonate-7-phosphate synthase
Rep1	Op0294r	1.12	**0.41**	1.03	0.50	0.69	*tyrB*	Rmet_1018	5021	4750	Q1LPM2	Aminotransferase
Rep1	Op0574r	1.12	**0.48**	1.06	0.67	0.73	*thrA*	Rmet_1966	2589	2580	Q1LLY1	Homoserine dehydrogenase
	**IIIB3. Lipid Metabolism**											
Rep1	Op0680r	0.93	**0.47**	0.85	0.77	0.93	-	Rmet_2419	308	64	Q1LKN0	Putative transmembrane protein
Rep1	Op0680r	0.76	**0.46**	0.94	0.69	0.81	*fabF*	Rmet_2426	2366	2708	Q1LKM3	3-oxoacyl-[acyl-carrier-protein] synthase II
Rep1	Op0680r	0.70	**0.31**	0.79	0.64	0.99	*acpP*	Rmet_2427	7863	7231	Q1LKM2	Acyl carrier protein
	**IIIB4. Carbohydrates**											
Rep1	Op0072r	0.68	**0.32**	0.77	0.58	1.13	*gpmA*	Rmet_0251	1132	1489	Q1LRT9	Phosphoglycerate mutase
Rep1	Op0840r	1.12	**0.38**	1.19	0.58	0.67	*cbbG2*	Rmet_2979	3058	3444	Q1LJ24	Glyceraldehyde-3-phosphate dehydrogenase
	**IIIB5. Metabolism, other**											
Rep1	Op0132r	0.90	**0.41**	0.94	0.52	0.94	*rfaF*	Rmet_0491	484	101	Q1LR49	Lipopolysaccharide heptosyltransferase II
Rep1	Op0135f	1.05	**0.49**	1.03	0.62	0.65	*purK*	Rmet_0506	1317	1474	Q1LR34	Phosphoribosylaminoimidazole carboxylase
Rep1	Op0772r	1.29	**0.49**	0.93	**0.48**	0.79	*pyrB*	Rmet_2740	1099	1152	Q1LJR3	Aspartate carbamoyltransferase
Rep1	Op0832r	1.04	**0.48**	1.01	0.80	0.84	*purB*	Rmet_2956	2277	2257	Q1LJ47	Adenylosuccinate lyase
Rep1	Op0870r	0.60	**0.44**	0.63	0.72	1.41	*nrdA*	Rmet_3088	1801	1926	Q1LIR6	Ribonucleoside-diphosphate reductase
Rep2	Op1128f	1.13	*0.37*	1.13	0.69	0.62	*pobA*	Rmet_4018	NF/NF	NF/NF	Q1LG40	4-hydroxybenzoate 3-monooxygenase
**IIIC. Environmental Information Processing**												
	**IIIC1. Membrane Transport**											
Rep1	Op0352r	0.58	**0.29**	1.14	1.22	0.73	-	Rmet_1210	NeF	NeF	Q1LP30	Inner-membrane translocator
Rep1	Op0352r	0.54	**0.22**	1.28	1.09	0.66	-	Rmet_1211	5080	6638	Q1LP29	Putative uncharacterized protein
Rep1	Op0614r	0.85	**0.46**	0.85	0.82	0.87	-	Rmet_2122	1618	866	Q1LLH5	Extracellular solute-binding protein, family 3
Rep1	Op0202r	0.91	**0.33**	1.03	0.78	0.82	-	Rmet_0712	4696	2666	Q1LQH8	OmpA/MotB
Rep1	Op0974r	0.79	**0.46**	1.06	0.74	1.39	-	Rmet_3518	1829	2120	Q1LHI6	Extracellular ligand-binding receptor
**IIID. Cellular Processes**												
Rep1	Op0862r	0.81	**0.23**	1.00	0.71	1.03	-	Rmet_3071	973	1262	Q1LIT3	Histone H1-like protein
Rep1	Op0862r	0.91	**0.39**	0.76	0.67	1.09	-	Rmet_3072	NeF	NeF	Q1LIT2	Putative uncharacterized protein
Rep1	Op0808r	0.87	**0.44**	0.69	0.64	1.08	*scd*	Rmet_2875	83	NF/NF	Q1LJC8	Fatty acid desaturase
Rep1	Op0878r	0.90	**0.48**	0.97	0.88	0.81	-	Rmet_3123	1518	1852	Q1LIN1	Cell division protein ftsZ
Rep2	Op1025r	0.90	**0.38**	1.08	0.93	1.04	-	Rmet_3677	3412	4268	Q1LH27	Nucleoid protein H-NS
Rep1	Op0608r	1.07	**0.49**	1.10	0.91	0.78	*hfq*	Rmet_2101	115	176	Q1LLJ6	Protein hfq
Rep2	Op1375r	1.02	**0.41**	1.22	0.81	0.96	-	Rmet_4742	8265	8706	Q1LE22	Histone-like DNA-binding protein
Rep1	Op0972r	1.41	**0.39**	1.65	0.51	0.69	-	Rmet_3503	681	477	Q1LHK1	Chromosome segregation DNA-binding protein
**IIIE. Other Processes**												
Rep1	Op0756r	0.83	**0.45**	0.94	0.84	0.81	-	Rmet_2673	896	849	Q1LJY0	TPR repeat
Rep1	Op0756r	0.86	**0.47**	0.90	0.74	0.80	-	Rmet_2674	2796	1461	Q1LJX9	OmpA/MotB
Rep1	Op0756r	0.72	**0.47**	0.83	0.59	0.91	*tolB*	Rmet_2675	1322	903	Q1LJX8	Protein tolB
Rep1	Op0022r	0.75	**0.40**	0.76	0.62	1.13	-	Rmet_0077	782	734	Q1LSB3	Putative uncharacterized protein
Rep1	Op0066r	0.93	**0.49**	0.88	0.76	0.82	-	Rmet_0224	184	532	Q1LRW6	Putative uncharacterized protein
Rep1	Op0066r	1.07	**0.43**	0.78	0.58	0.69	-	Rmet_0227	NeF	NeF	Q1LRW3	Ferredoxin-like protein

Rep1	Op0140r	1.07	0.45	1.12	0.75	0.90	-	Rmet_0521	13246	11923	Q1LR19 Uncharacterized protein UPF0065
Rep1	Op0181f	1.01	0.38	0.86	0.97	1.01	-	Rmet_0655	NeF	NeF	Q1LQN5 Putative membrane protein
Rep1	Op0234r	0.90	0.45	0.65	0.45	0.72	-	Rmet_0825	507	187	Q1LQ65 Putative uncharacterized protein
Rep1	Op0244r	1.37	0.37	1.75	0.42	0.54	-	Rmet_0854	173	69	Q1LQ36 Serine/threonine protein kinase
Rep1	Op0312r	1.00	0.47	1.09	0.49	0.68	-	Rmet_1084	604	473	Q1LPF6 Tetratricopeptide TPR_2
Rep1	Op0328r	0.82	0.38	0.70	0.67	0.92	-	Rmet_1146	2133	1776	Q1LP94 Electron transfer flavoprotein-ubiquinone oxidoreductase
Rep1	Op0348r	1.00	0.43	1.01	0.53	1.78	-	Rmet_1200	2694	2115	Q1LP40 Phasin
Rep1	Op0462r	0.87	0.42	0.97	0.84	0.97	-	Rmet_1642	85	NF/NF	Q1LMV1 Putative uncharacterized protein
Rep1	Op0571f	0.87	0.33	0.83	1.03	1.05	-	Rmet_1949	NeF	NeF	Q1LLZ8 General substrate transporter
Rep1	Op0612r	0.72	0.38	0.97	0.88	1.01	-	Rmet_2116	1079	548	Q1LLI1 Peptidase M23B
Rep1	Op0618r	0.92	0.49	1.00	0.85	0.72	-	Rmet_2142	NeF	NeF	Q1LLF5 NLP/P60
Rep1	Op0652r	1.05	0.45	1.30	0.72	3.26	-	Rmet_2281	4287	5000	Q1LL16 Putative uncharacterized protein
Rep1	Op0654r	1.40	0.47	0.93	0.63	0.75	-	Rmet_2286	0	0	Q1LL11 Putative uncharacterized protein
Rep1	Op0659f	1.77	0.14	1.30	0.49	0.78	-	Rmet_2305	NeF	NeF	Q8GQ16 Putative uncharacterized protein ORF C95
Rep1	Op0694r	0.58	0.35	0.89	0.70	1.43	-	Rmet_2502	300	243	Q1LKE7 Cytochrome c, class I
Rep1	Op0701f	0.64	0.48	1.16	0.73	0.80	-	Rmet_2539	NeF	NeF	Q1LKB0 Putative uncharacterized protein
Rep1	Op0725f	0.51	0.28	0.65	0.92	1.75	-	Rmet_2593	24	NF/NF	Q1LK56 Glycosyl transferase, family 28
Rep1	Op0760r	0.89	0.43	0.79	0.68	0.80	-	Rmet_2697	NeF	NeF	Q1LJV6 Lytic transglycosylase, catalytic
Rep1	Op0818r	0.89	0.44	1.15	0.87	1.05	-	Rmet_2902	1566	1874	Q1LJA1 Ornithine carbamoyltransferase
Rep1	Op0940r	0.79	0.27	0.67	1.00	1.31	-	Rmet_3379	543	NF/NF	Q1LHX5 Transcriptional regulator, AsnC family
Rep1	Op0992r	1.00	0.38	1.13	0.59	0.90	-	Rmet_3572	NeF	NeF	Q1LHD2 17 kDa surface antigen
Rep2	Op1050f	0.98	0.24	0.91	1.04	0.89	-	Rmet_3779	NeF	NeF	Q1LGS7 Monooxygenase, FAD-binding
Rep2	Op1067r	0.65	0.13	2.92	0.28	0.38	-	Rmet_3842	NeF	NeF	Q1LGL6 Putative uncharacterized protein
Rep2	Op1419r	1.20	0.18	0.89	0.51	1.06	-	Rmet_4864	NeF	NeF	Q1LDQ0 Putative uncharacterized protein
Rep2	Op1448f	0.88	0.16	0.92	0.90	0.88	-	Rmet_4934	NeF	NeF	Q1LDI0 Xanthine/uracil/vitamin C permease
Rep2	Op1493r	0.73	0.40	0.88	0.76	0.96	*metE*	Rmet_5073	3925	4886	Q1LD41 Putative uncharacterized protein
Rep2	Op1641r	0.91	0.48	0.86	0.81	0.92	-	Rmet_5519	136	96	Q1LBU8 Electron transfer flavoprotein beta-subunit

IV. Not regulated in Q(EDTA:Δ7/ΔzupT) AND up-regulated in Q(Zn:Δ7/ΔzupT)

Rep1	Op0003f	1.72	2.26	1.36	1.42	0.65	-	Rmet_0014	NeF	NeF	Q1LSH6 Putative uncharacterized protein
Rep1	Op0094r	1.06	2.53	1.06	2.01	0.86	-	Rmet_0332	25	NF/NF	Q1LRK8 Glyoxalase/bleomycin resistance protein/dioxygenase
Rep1	Op0098r	1.00	2.09	0.97	1.12	0.98	-	Rmet_0349	384	352	Q1LRJ1 ABC transporter-related protein
Rep1	Op0164r	1.00	2.73	0.85	2.05	0.93	-	Rmet_0581	NeF	NeF	Q1LQV9 Paraquat-inducible protein A
Rep1	Op0187f	1.59	2.08	1.16	1.24	0.97	*pilI*	Rmet_0671	66	NF/NF	Q1LQL9 CheW protein
Rep1	Op0197f	1.26	2.33	1.42	1.63	0.83	*gltI*	Rmet_0701	524	225	Q1LQI9 Extracellular solute-binding protein, family 3
Rep1	Op0267f	1.61	2.99	1.24	0.91	0.32	*livF9*	Rmet_0956	171	308	Q1LPT4 ABC transporter-related protein
Rep1	Op0267f	1.04	2.68	0.96	2.42	1.04	-	Rmet_0957	NeF	NeF	Q1LPT3 Urease accessory protein ureD
Rep1	Op0379f	1.65	2.01	1.45	1.12	0.72	*traG*	Rmet_1339	NeF	NeF	Q1LNQ4 TRAG protein
Rep1	Op0379f	1.66	2.85	1.27	0.91	0.35	-	Rmet_1346	NeF	NeF	Q1LNP7 Putative lipoprotein
Rep1	Op0385f	1.29	2.21	1.17	1.09	0.87	-	Rmet_1381	48	215	Q1LNL2 Transcriptional regulator, LytR/AlgR family
Rep1	Op0452r	1.34	2.16	1.29	0.90	0.66	-	Rmet_1624	NeF	NeF	Q1LMW9 Drug resistance transporter EmrB/QacA subfamily
Rep1	Op0481f	0.76	2.76	1.39	1.38	8.04	-	Rmet_1705	1360	576	Q1LMP0 Putative uncharacterized protein
Rep1	Op0483f	0.65	2.52	1.05	1.20	2.41	-	Rmet_1708	NeF	NeF	Q1LMN7 Putative uncharacterized protein
Rep1	Op0539f	1.42	2.03	1.54	1.16	0.51	-	Rmet_1867	412	438	Q1LM80 Transcriptional regulator BolA
Rep1	Op0623f	1.68	2.25	1.52	1.98	1.06	-	Rmet_2163	203	163	Q1LLD4 Transcriptional regulator, XRE family
Rep1	Op0663f	1.24	3.67	0.46	1.92	1.49	-	Rmet_2360	NeF	NeF	Q8GQ70 Putative uncharacterized protein ORF C40
Rep1	Op0737f	1.63	2.44	1.35	1.18	0.45	*valS*	Rmet_2630	1578	1707	Q1LK19 Valyl-tRNA synthetase
Rep1	Op0775f	1.10	2.04	0.98	1.74	1.22	-	Rmet_2752	232	82	Q1LJQ1 Tripartite ATP-independent periplasmic transporter, DctQ component
Rep2	Op1020f	1.10	2.62	1.60	1.80	1.13	-	Rmet_3666	NeF	NeF	Q1LH38 Putative uncharacterized protein
Rep2	Op1148f	1.67	2.36	1.14	1.21	0.95	*phoA1*	Rmet_4084	1248	137	Q1LFX5 Alkaline phosphatase

Rep2	Op1162f	1.62	**2.11**	1.32	1.69	1.06	-	Rmet_4126	NeF	NeF	Q1LFT3 Nitrogen fixation protein NifQ, putative
Rep2	Op1330f	1.81	**2.23**	0.89	1.19	0.96	-	Rmet_4615	NeF	NeF	Q1LEE9 Transcriptional regulator, GntR family
Rep2	Op1375r	1.12	*2.48*	1.13	1.96	0.73	-	Rmet_4743	NeF	NeF	Q1LE21 Putative uncharacterized protein
Rep2	Op1412f	1.36	**2.50**	1.33	1.97	0.82	-	Rmet_4843	16	13	Q1LDS1 ABC transporter related
Rep2	Op1490f	1.15	**2.10**	1.14	1.56	0.95	-	Rmet_5058	NF/NF	NF/NF	Q1LD56 Uncharacterized protein UPF0065
Rep2	Op1490f	0.98	**2.54**	1.02	1.83	1.08	*atoD*	Rmet_5059	22	NF/NF	Q1LD55 3-oxoacid CoA-transferase, subunit A
Rep2	Op1490f	1.37	**2.89**	1.19	**2.06**	0.88	*atoA1*	Rmet_5060	NeF	NeF	Q1LD54 Butyryl-CoA:acetate CoA transferase
Rep2	Op1516f	1.87	**2.03**	1.66	1.27	0.69	-	Rmet_5151	NeF	NeF	Q1LCW6 Transcriptional regulator, LysR family
Rep2	Op1552f	1.65	**2.34**	1.36	0.60	**0.23**	*fliF*	Rmet_5261	NeF	NeF	Q1LCK6 Flagellar M-ring protein FliF
Rep2	Op1644f	1.30	**2.07**	1.01	1.13	0.65	-	Rmet_5531	351	231	Q1LBT6 Transcriptional regulator, GntR family
Rep2	Op1644f	1.36	**2.43**	1.13	1.16	0.60	-	Rmet_5532	408	364	Q1LBT5 Transcriptional regulator, GntR family
Rep2	Op1701r	1.03	*2.17*	1.06	*2.14*	0.86	-	Rmet_5694	NeF	NeF	Q1LBC3 Putative uncharacterized protein

V. Not regulated in Q(EDTA:Δ7/ΔzupT) AND not regulated in Q(Zn:Δ7/ΔzupT)

VA. Down-regulated in Q(EDTA:Δ7/AE104) AND down-regulated in Q(Zn:Δ7/AE104)

Rep1	Op0026r	1.39	1.01	**0.25**	**0.50**	**2.66**	-	Rmet_0092	46	NF/NF	Q1LS98 Histidine kinase
Rep1	Op0228r	1.81	1.18	**0.22**	**0.27**	**2.12**	-	Rmet_0804	NeF	NeF	Q1LQ86 SPFH domain, Band 7 family protein
Rep1	Op0228r	1.60	1.10	**0.29**	**0.35**	**2.21**	-	Rmet_0805	NeF	NeF	Q1LQ85 Putative uncharacterized protein
Rep1	Op0248r	1.29	0.66	**0.27**	**0.41**	**2.36**	-	Rmet_0863	NeF	NeF	Q1LQ27 Putative uncharacterized protein
Rep1	Op0249f	1.36	1.59	**0.33**	**0.43**	**2.30**	-	Rmet_0864	135	156	Q1LQ26 Putative uncharacterized protein
Rep1	Op0249f	1.21	1.77	**0.28**	**0.38**	**2.67**	-	Rmet_0865	NeF	NeF	Q1LQ25 Putative uncharacterized protein
Rep1	Op0638r	1.74	1.11	**0.49**	**0.41**	1.40	-	Rmet_2231	NeF	NeF	Q1LL66 Binding-protein-dependent transport systems inner membrane component
Rep1	Op0664r	1.40	0.59	**0.17**	**0.16**	1.96	-	Rmet_2362	30	NF/NF	Q8GQ72 Putative uncharacterized protein ORF C38
Rep1	Op0748r	1.06	1.17	**0.31**	**0.47**	1.62	-	Rmet_2647	NF/NF	NF/NF	Q1LK02 Transcriptional regulator, AsnC family
Rep1	Op0808r	1.62	1.26	**0.22**	**0.38**	**2.68**	-	Rmet_2876	105	NF/NF	Q1LJC7 MscS Mechanosensitive ion channel
Rep1	Op0935f	0.70	0.73	**0.13**	**0.26**	**2.29**	-	Rmet_3358	69	55	Q1LHZ6 Putative uncharacterized protein
Rep1	Op0935f	0.65	0.66	**0.16**	**0.36**	**2.85**	-	Rmet_3360	NeF	NeF	Q1LHZ4 Putative uncharacterized protein
Rep1	Op0935f	0.72	0.89	**0.20**	**0.35**	1.96	-	Rmet_3361	NeF	NeF	Q1LHZ3 Putative uncharacterized protein
Rep1	Op0935f	0.94	0.67	**0.26**	**0.27**	1.81	-	Rmet_3362	NeF	NeF	Q1LHZ2 Putative uncharacterized protein
Rep1	Op0936r	0.85	0.98	**0.15**	**0.20**	**2.11**	-	Rmet_3365	NeF	NeF	Q1LHY9 Integrase, catalytic region
Rep2	Op1009r	1.53	1.22	**0.27**	**0.30**	1.87	-	Rmet_3625	249	96	Q1LH79 Putative uncharacterized protein
Rep2	Op1275r	1.30	0.95	**0.47**	**0.46**	1.74	-	Rmet_4472	NeF	NeF	Q1LEG4 Sulphate transporter
Rep2	Op1279r	1.98	0.98	**0.46**	**0.38**	1.84	-	Rmet_4494	23	255	Q1LER7 Putative uncharacterized protein
Rep2	Op1324f	1.35	1.21	**0.36**	**0.50**	1.97	-	Rmet_4600	NeF	NeF	Q1LEG4 Sulphate transporter
Rep2	Op1341r	1.43	1.41	**0.18**	**0.36**	**3.04**	-	Rmet_4645	157	NF/NF	Q1LEB9 Uncharacterized protein UPF0065
Rep2	Op1461r	1.32	0.98	**0.24**	**0.40**	**2.38**	-	Rmet_4978	NQ/NQ	NQ/NQ	Q1LDD6 Two component transcriptional regulator, winged helix family
Rep2	Op1505r	1.25	1.35	**0.32**	**0.43**	1.84	-	Rmet_5112	NF/NF	NF/NF	Q1LD05 Putative uncharacterized protein

VB. Down-regulated in Q(EDTA:Δ7/AE104) AND not regulated in Q(Zn:Δ7/AE104)

Rep1	Op0004r	1.17	1.14	**0.37**	0.75	**2.12**	-	Rmet_0022	29	NF/NF	Q1LSG8 Transcriptional regulator, AraC family
Rep1	Op0027f	1.29	1.17	**0.49**	0.83	**2.21**	-	Rmet_0093	NeF	NeF	Q1LS97 UPF0391 membrane protein Rmet_0093
Rep1	Op0028r	1.58	1.05	**0.32**	0.51	**2.54**	-	Rmet_0095	652	947	Q1LS95 Putative uncharacterized protein
Rep1	Op0102r	1.04	0.72	**0.26**	0.56	**2.33**	-	Rmet_0360	21	27	Q1LRI0 Carbon monoxide dehydrogenase subunit G
Rep1	Op0102r	1.28	0.96	**0.37**	0.55	1.83	-	Rmet_0364	76	NF/NF	Q1LRH6 Xanthine dehydrogenase, molybdenum binding subunit apoprotein
Rep1	Op0112r	1.23	1.22	**0.48**	0.69	1.57	-	Rmet_0409	NeF	NeF	Q1LRD0 50S ribosomal protein L13
Rep1	Op0227f	1.28	0.71	**0.44**	0.67	1.37	-	Rmet_0799	270	262	Q1LQ91 Binding-protein-dependent transport systems inner membrane component
Rep1	Op0412r	0.95	0.65	**0.50**	0.62	1.36	-	Rmet_1472	758	136	Q1LNC1 KAP P-loop
Rep1	Op0412r	1.05	0.83	**0.43**	0.72	1.67	-	Rmet_1473	255	NF/NF	Q1LNC0 Retron reverse transcriptase
Rep1	Op0412r	1.10	1.18	**0.43**	0.99	1.56	-	Rmet_1474	NeF	NeF	Q1LNB9 Putative uncharacterized protein
Rep1	Op0489f	1.01	1.32	**0.47**	0.79	1.66	-	Rmet_1728	0	0	Q46NG6 General substrate transporter:Major facilitator superfamily MFS_1
Rep1	Op0577f	0.88	0.83	**0.49**	0.74	1.76	-	Rmet_1982	NF/NF	34	Q1LLW5 Putative uncharacterized protein

Rep1	Op0635f	1.21	1.26	**0.34**	0.55	1.71	-	Rmet_2218	141	NF/NF	Q1LL79 Luciferase-like protein
Rep1	Op0662r	0.85	0.90	**0.49**	0.87	1.55	-	Rmet_2342	249	24	A0H9A7 AAA ATPase, central region
Rep1	Op0662r	1.14	0.86	**0.45**	0.60	1.74	-	Rmet_2344	133	170	Q8GQ54 Putative excisionase
Rep1	Op0664r	0.96	0.85	**0.38**	0.60	1.86	-	Rmet_2363	189	218	Q8GQ73 Putative uncharacterized protein ORF C37
Rep1	Op0754r	1.28	1.09	**0.43**	0.60	1.88	-	Rmet_2658	0	0	Q0K7X0 Predicted signal transduction protein containingPAS, GGDEF and EAL domains
Rep1	Op0757f	1.18	0.81	**0.50**	0.56	1.36	-	Rmet_2683	257	NF/NF	Q1LJX0 Putative transmembrane protein
Rep1	Op0815f	0.92	0.77	**0.39**	0.71	1.71	-	Rmet_2897	31	NF/NF	Q1LJA6 Putative uncharacterized protein
Rep1	Op0868r	1.54	1.08	**0.38**	0.81	1.73	-	Rmet_3085	NeF	NeF	Q1LIR9 Alcohol dehydrogenase, zinc-binding
Rep1	Op0869f	1.27	1.05	**0.17**	0.52	**4.31**	-	Rmet_3086	135	151	Q1LIR8 Uncharacterized protein UPF0065
Rep1	Op0870r	1.17	1.05	**0.25**	0.63	**2.09**	*nrdB*	Rmet_3087	616	535	Q1LIR7 Ribonucleotide reductase
Rep1	Op0896r	0.98	0.99	**0.50**	0.63	1.46	-	Rmet_3188	48	NF/NF	Q1LIG6 Peptidase M61
Rep1	Op0950r	0.64	0.85	**0.46**	0.76	1.25	-	Rmet_3428	656	789	Q1LHS6 Transport-associated
Rep2	Op1007r	1.12	0.91	**0.45**	0.71	1.77	-	Rmet_3618	950	1189	Q1LH86 OsmC-like protein
Rep2	Op1007r	1.10	1.25	**0.29**	0.71	**2.66**	-	Rmet_3619	NeF	NeF	Q1LH85 Transcriptional regulator, MarR family
Rep2	Op1009r	1.08	1.04	**0.45**	0.66	1.70	-	Rmet_3623	NeF	NeF	Q1LH81 Transcriptional regulator, MarR family
Rep2	Op1160f	1.31	1.04	**0.41**	0.93	1.43	-	Rmet_4119	NeF	NeF	Q1LFU0 Putative uncharacterized protein
Rep2	Op1164f	1.23	1.22	**0.36**	0.61	1.82	-	Rmet_4128	NeF	NeF	Q1LFT1 Putative uncharacterized protein
Rep2	Op1190f	1.03	1.06	**0.40**	0.70	1.91	-	Rmet_4198	NeF	NeF	Q1LFL1 Transcriptional regulator, PadR family
Rep2	Op1279r	1.17	1.25	**0.39**	0.57	1.69	-	Rmet_4490	NeF	NeF	Q1LES1 Putative uncharacterized protein
Rep2	Op1279r	1.20	1.19	**0.30**	0.68	**2.67**	-	Rmet_4493	0	0	Q1LER8 Putative uncharacterized protein
Rep2	Op1341r	1.10	1.41	**0.36**	0.76	**2.04**	-	Rmet_4644	75	NF/NF	Q1LEC0 Carboxymuconolactone decarboxylase
Rep2	Op1425r	0.91	1.09	**0.45**	0.68	1.59	-	Rmet_4876	34	NF/NF	Q1LDN8 Uncharacterized protein UPF0065
Rep2	Op1461r	1.19	1.08	**0.43**	0.61	1.70	-	Rmet_4979	NeF	NeF	Q1LDD5 Putative uncharacterized protein
Rep2	Op1513r	0.96	1.26	**0.41**	0.80	**2.04**	-	Rmet_5133	32	91	Q1LCY4 Acyl-CoA dehydrogenase-like protein
Rep2	Op1579r	1.37	1.51	*0.49*	0.57	0.63	-	Rmet_5333	NeF	NeF	Q1LCD4 Putative uncharacterized protein
Rep2	Op1590f	0.81	1.17	**0.50**	0.84	1.74	-	Rmet_5352	NeF	NeF	Q1LCB5 Putative uncharacterized protein
Rep2	Op1645r	1.22	1.42	**0.38**	0.52	1.31	-	Rmet_5533	NeF	NeF	Q1LBT4 Nitroreductase
Rep2	Op1645r	0.77	0.87	**0.47**	0.66	1.65	-	Rmet_5534	NeF	NeF	Q1LBT3 2-nitropropane dioxygenase, NPD
	**VC. Up-regulated in Q(EDTA:Δ7/AE104) AND Up-regulated in Q(Zn:Δ7/AE104)**										
Rep1	Op0032r	1.32	1.09	*2.09*	*0.42*	0.73	*zur*	Rmet_0128	77	102	Q1LS62 Putative ferric uptake regulator, FUR family
		**aut-region 1**									
Rep1	Op0421f	0.98	0.95	**2.46**	**2.22**	1.02	-	Rmet_1491	154	NF/NF	Q6SKD6 Transposase
Rep1	Op0422r	0.93	0.79	**3.17**	**2.00**	0.95	-	Rmet_1498	NF/NF	169	Q1LN95 von Willebrand factor, type A
Rep1	Op0422r	1.08	0.86	**5.10**	**2.68**	0.96	*cbbQ*	Rmet_1499	NF/NF	433	Q1LN94 ATPase associated with various cellular activities, AAA_5
Rep1	Op0422r	0.86	0.66	**2.42**	1.49	0.97	*cbbS*	Rmet_1500	NF/NF	1689	Q1LN93 Ribulose bisphosphate carboxylase small chain
Rep1	Op0422r	0.90	0.66	**2.41**	1.63	1.09	*rbcL*	Rmet_1501	70	6269	Q1LN92 Ribulose bisphosphate carboxylase large chain
Rep1	Op0423f	1.28	1.33	**5.86**	**8.53**	1.26	-	Rmet_1502	NF/NF	290	Q1LN91 Transcriptional regulator, LysR family
Rep1	Op0423f	1.07	1.08	**2.27**	**2.27**	1.00	-	Rmet_1503	NF/NF	NF/NQ	Q9RBF5 Ralstonia eutropha insertion sequence IS1090
Rep1	Op0424r	1.04	1.10	**2.56**	**2.84**	1.12	-	Rmet_1505	0	0	Q1LN88 ISSod11, transposase
Rep1	Op0425f	1.75	0.90	**6.87**	**5.60**	0.64	*cbbF2*	Rmet_1511	NF/NF	237	Q1LN82 D-fructose 1,6-bisphosphatase
Rep1	Op0425f	1.42	1.27	**2.26**	**2.27**	0.83	*cbbP*	Rmet_1512	NF/NF	531	Q1LN81 Phosphoribulokinase
Rep1	Op0425f	1.54	1.41	**3.54**	**2.71**	0.63	*cbbT1*	Rmet_1513	NF/NF	327	Q1LN80 Transketolase
Rep1	Op0425f	1.48	1.14	**3.50**	**2.81**	0.79	*cbbZ1*	Rmet_1514	163	120	Q1LN79 Phosphoglycolate phosphatase
Rep1	Op0425f	1.68	1.16	**3.09**	**2.41**	0.85	*cbbG1*	Rmet_1515	62	573	Q1LN78 Glyceraldehyde-3-phosphate dehydrogenase
Rep1	Op0425f	1.83	1.00	**6.90**	**2.48**	*0.47*	-	Rmet_1517	NF/NF	61	Q1LN76 HAD-superfamily hydrolase subfamily IA, variant 3
Rep1	Op0425f	1.30	1.40	**3.36**	**3.39**	0.93	*cbbA2*	Rmet_1518	68	191	Q1LN75 Fructose-bisphosphate aldolase
Rep1	Op0425f	1.25	1.14	**2.04**	**2.23**	1.06	*pykA2*	Rmet_1519	NeF	NeF	Q1LN74 Pyruvate kinase
Rep1	Op0425f	1.38	0.97	**2.44**	**2.48**	0.96	*cbbJ1*	Rmet_1520	NF/NF	101	Q1LN73 Triosephosphate isomerase
Rep1	Op0425f	1.49	1.23	**6.39**	**5.92**	0.81	*cbbI1*	Rmet_1521	NF/NF	76	Q1LN72 Ribose-5-phosphate isomerase
Rep1	Op0425f	1.25	1.26	**5.32**	**7.88**	1.19	*hoxF*	Rmet_1522	NF/NF	1694	Q1LN71 Respiratory-chain NADH dehydrogenase domain, 51 kDa subunit
Rep1	Op0425f	1.12	1.30	**9.79**	**13.64**	1.36	*hoxU*	Rmet_1523	NF/NF	1098	Q1LN70 Ferredoxin

Rep1	Op0425f	0.93	1.23	**9.52**	**16.24**	1.64	*hoxY*	Rmet_1524	NF/NF	1855	Q1LN69 NADH ubiquinone oxidoreductase, 20 kDa subunit
Rep1	Op0425f	1.15	1.38	**11.69**	**16.24**	1.34	*hoxH*	Rmet_1525	34	4611	Q1LN68 Nickel-dependent hydrogenase, large subunit
Rep1	Op0425f	0.96	1.21	**9.28**	**15.52**	1.62	-	Rmet_1526	NF/NF	1766	Q1LN67 HoxW protein
Rep1	Op0425f	1.25	1.19	**10.69**	**15.53**	1.30	-	Rmet_1527	NF/NF	8605	Q1LN66 Cyclic nucleotide-binding domain (CNMP-BD) protein
Rep1	Op0425f	1.36	1.21	**4.83**	**7.60**	1.20	-	Rmet_1528	0	0	Q1LN65 Alanine dehydrogenase/PNT-like protein
Rep1	Op0426r	1.21	1.26	**2.48**	**4.58**	1.19	-	Rmet_1529	NF/NF	373	Q1LB62 Integrase, catalytic region
Rep1	Op0427f	1.28	1.11	**2.53**	**2.04**	0.85	-	Rmet_1530	0	0	Q1LN63 Alanine dehydrogenase/PNT-like protein
Rep1	Op0427f	0.89	0.95	**6.59**	**15.93**	1.85	-	Rmet_1534	0	0	Q1LN59 Putative uncharacterized protein
Rep1	Op0427f	0.95	0.91	**7.30**	**16.19**	1.77	*hypA2*	Rmet_1535	NF/NF	622	Q1LN58 Hydrogenase nickel insertion protein HypA
Rep1	Op0427f	0.81	0.62	**6.92**	**9.84**	1.49	*hypB2*	Rmet_1536	NF/NF	5295	Q1LN57 Hydrogenase accessory protein HypB
Rep1	Op0427f	1.13	1.04	**8.42**	**14.59**	1.54	*hypF2*	Rmet_1537	NF/NF	504	Q1LN56 (NiFe) hydrogenase maturation protein HypF
Rep1	Op0427f	1.11	0.96	**7.03**	**11.99**	1.41	*hpyC2*	Rmet_1538	NeF	NeF	Q1LN55 Hydrogenase assembly chaperone hypC/hupF
Rep1	Op0427f	1.03	0.80	**7.41**	**13.77**	1.67	*hypD2*	Rmet_1539	NF/NF	493	Q1LN54 Hydrogenase expression/formation protein HypD
Rep1	Op0427f	0.97	0.85	**7.43**	**15.10**	1.77	*hypE2*	Rmet_1540	NF/NF	2965	Q1LN53 Hydrogenase expression/formation protein HypE
Rep1	Op0427f	1.01	0.87	**7.31**	**14.07**	1.72	*hoxX*	Rmet_1541	NF/NF	371	Q1LN52 Formyl transferase-like protein
Rep1	Op0427f	1.18	1.10	**8.24**	**17.07**	1.61	*hoxA'*	Rmet_1542	608	240	Q1LN51 Response regulator receiver domain protein (CheY-like)
Rep1	Op0428r	1.25	1.25	**2.83**	**3.97**	1.19	-	Rmet_1543	NF/NQ	NF/NF	Q1LN50 Stress responsive alpha-beta barrel
Rep1	Op0430r	1.04	1.21	**2.56**	**2.43**	0.78	-	Rmet_1545	NeF	NeF	Q1LN48 Putative uncharacterized protein
Rep2	Op1724f	1.35	1.32	**3.39**	**5.08**	1.17	-	Rmet_5754	0	0	Q1LB63 Fumarate hydratase, class II
	**VD. Not regulated in Q(EDTA:Δ7/AE104) AND Up-regulated in Q(Zn:Δ7/AE104)**										
Rep1	Op0397f	0.91	1.66	1.58	**2.12**	1.52	-	Rmet_1418	133	108	Q1LNH5 Putative integral membrane protein
Rep1	Op0425f	1.30	0.99	1.85	**2.62**	1.02	*cbbE1*	Rmet_1510	NF/NF	32	Q1LN83 Ribulose-5-phosphate 3-epimerase
Rep1	Op0431f	0.93	1.26	1.79	**2.38**	1.08	-	Rmet_1546	NeF	NeF	Q1LN47 Transcriptional regulator, LysR family
Rep1	Op0813f	1.17	1.78	1.15	**2.07**	1.26	-	Rmet_2894	962	840	Q1LJA9 Cold-shock DNA-binding protein family
Rep2	Op1091r	1.31	1.86	1.12	**2.25**	1.19	-	Rmet_3903	NeF	NeF	Q1LGF5 Putative uncharacterized protein
Rep2	Op1259r	1.24	1.31	1.89	**2.40**	0.85	-	Rmet_4417	0	0	Q1LEZ4 Uncharacterized protein UPF0065
	**VE. Not regulated in Q(EDTA:Δ7/AE104) AND down-regulated in Q(Zn:Δ7/AE104)**										
Rep1	Op0047f	1.85	1.08	1.53	*0.49*	0.78	*betA1*	Rmet_0159	NeF	NeF	Q1LS31 Glucose-methanol-choline oxidoreductase
Rep1	Op0098r	1.99	0.99	1.60	*0.45*	0.55	-	Rmet_0351	469	14	Q1LRI9 Transcriptional regulator, TetR family
Rep1	Op0226r	1.69	1.15	1.06	*0.43*	0.73	-	Rmet_0795	NeF	NeF	Q1LQ95 Inner-membrane translocator
Rep1	Op0228r	1.34	0.91	0.51	**0.46**	1.17	-	Rmet_0803	NeF	NeF	Q1LQ87 Putative uncharacterized protein
Rep1	Op0490r	1.44	1.18	1.19	*0.49*	0.59	-	Rmet_1731	64	NF/NF	Q1LML6 Putative uncharacterized protein
Rep1	Op0506r	1.57	0.94	1.47	*0.49*	0.60	-	Rmet_1765	214	NF/NF	Q1LMI2 Putative uncharacterized protein
Rep1	Op0655f	1.48	1.32	1.45	*0.49*	0.55	-	Rmet_2293	NF/NF	117	Q8GQ05 Putative uncharacterized protein ORF C106
Rep1	Op0655f	1.84	0.74	1.42	*0.49*	0.65	-	Rmet_2294	53	NF/NF	Q8GQ06 Putative uncharacterized protein ORF C105
Rep1	Op0661f	1.90	1.14	1.66	*0.47*	0.55	-	Rmet_2322	NeF	NeF	Q1LKX7 Putative uncharacterized protein
Rep1	Op0662r	1.77	0.84	0.67	**0.49**	1.36	-	Rmet_2343	43	105	Q8GQ53 Putative uncharacterized protein ORF C57
Rep1	Op0663f	1.43	0.96	0.88	*0.45*	0.80	-	Rmet_2354	NeF	NeF	Q8GQ64 Putative uncharacterized protein ORF C46
Rep1	Op0701f	1.62	1.08	1.40	*0.40*	*0.48*	-	Rmet_2536	NeF	NeF	Q1LKB3 L-carnitine dehydratase/bile acid-inducible protein F
Rep1	Op0709f	1.08	0.54	0.60	**0.46**	0.59	-	Rmet_2564	418	593	Q1LK85 DEAD/DEAH box helicase-like protein
Rep1	Op0730r	1.57	0.61	1.46	**0.49**	0.60	*rpsU2*	Rmet_2609	160	119	Q1LK40 30S ribosomal protein S21 2
Rep1	Op0784r	1.77	0.63	1.47	*0.46*	0.53	*cobT*	Rmet_2785	317	340	Q1LJL8 Nicotinate-nucleotide-dimethylbenzimidazole phosphoribosyltransferase
Rep1	Op0804r	1.35	0.70	1.59	*0.39*	0.69	*hutI*	Rmet_2852	NeF	NeF	Q1LJF1 Imidazolonepropionase
Rep1	Op0836r	1.84	0.79	1.75	*0.44*	0.50	-	Rmet_2969	79	NF/NF	Q1LJ34 Putative uncharacterized protein
Rep1	Op0846r	1.63	1.09	1.53	*0.49*	0.57	*hmzB*	Rmet_3012	NeF	NeF	Q1LIZ1 Secretion protein HlyD
Rep1	Op0974r	1.94	1.11	1.73	*0.44*	*0.50*	-	Rmet_3514	NeF	NeF	Q1LHJ0 Putative uncharacterized protein
Rep1	Op0987f	1.91	1.16	1.59	*0.48*	0.54	-	Rmet_3558	NeF	NeF	Q1LHE6 Na+/solute symporter
Rep2	Op1073r	1.93	1.05	1.76	*0.45*	*0.48*	-	Rmet_3862	NeF	NeF	Q1LGJ6 Lysine exporter protein (LYSE/YGGA)
Rep2	Op1115r	1.69	1.31	1.19	*0.48*	0.70	*livM4*	Rmet_3979	NeF	NeF	Q1LG79 Inner-membrane translocator
Rep2	Op1169r	1.74	1.09	1.46	*0.47*	0.62	-	Rmet_4145	455	418	Q1LFR4 L-carnitine dehydratase/bile acid-inducible protein F
Rep2	Op1175r	1.68	0.68	1.51	*0.45*	0.55	-	Rmet_4171	103	21	Q1LFN8 Glycosyl transferase, family 2

Rep2	Op1207r	1.74	1.27	1.61	0.45	0.54	-	Rmet_4260	NeF	NeF	Q1LFF0 Pirin-like protein
Rep2	Op1217r	1.96	0.91	1.46	0.42	0.68	-	Rmet_4283	NeF	NeF	Q1LFC7 Putative uncharacterized protein
Rep2	Op1276f	1.80	0.99	1.50	0.44	0.51	-	Rmet_4473	NF/NF	NF/NF	Q7X3A9 Putative resolvase
Rep2	Op1297r	1.75	1.14	1.76	0.43	0.66	-	Rmet_4532	NeF	NeF	Q1LEM9 von Willebrand factor, type A
Rep2	Op1303r	1.85	1.05	1.78	0.49	0.50	-	Rmet_4547	30	NF/NF	Q1LEL4 Putative outer membrane porin signal peptide protein
Rep2	Op1419r	1.46	1.11	1.25	0.46	0.56	-	Rmet_4865	NeF	NeF	Q1LDP9 RND efflux system, outer membrane lipoprotein, NodT
Rep2	Op1457r	1.55	0.93	1.16	**0.46**	0.77	-	Rmet_4960	147	100	Q1LDF4 Pili assembly chaperone
Rep2	Op1614f	1.31	1.08	0.78	**0.42**	0.65	-	Rmet_5426	NeF	NeF	Q1LC41 Putative uncharacterized protein
Rep2	Op1619r	1.94	1.08	1.85	0.48	0.52	-	Rmet_5444	NeF	NeF	Q1LC23 FAD dependent oxidoreductase
Rep2	Op1747r	1.92	0.91	1.85	0.45	0.45	-	Rmet_5800	173	295	Q1LB17 6-phosphogluconolactonase
Rep2	Op1777r	1.71	1.00	1.45	0.41	0.62	*leuD*	Rmet_5867	NeF	NeF	Q1LAV0 3-isopropylmalate dehydratase, small subunit
Rep2	Op1792f	1.54	0.87	1.54	0.47	0.58	-	Rmet_5907	NeF	NeF	Q1LAR0 Putative uncharacterized protein

VF. Not regulated in Q(EDTA:Δ7/AE104) AND not regulated in Q(Zn:Δ7/AE104)

VF1. Down-regulated in Q(AE104:EDTA/Zn)

Rep1	Op0031f	1.29	0.95	1.11	0.59	**0.28**	*bioA*	Rmet_0114	1808	1704	Q1LS76 Adenosylmethionine-8-amino-7-oxononanoate aminotransferase
Rep1	Op0031f	1.37	1.01	1.16	0.64	**0.26**	*bioF*	Rmet_0115	1589	1446	Q1LS75 8-amino-7-oxononanoate synthase
Rep1	Op0031f	1.32	1.11	1.14	0.66	**0.24**	*bioD*	Rmet_0116	1534	1546	Q1LS74 Dethiobiotin synthase
Rep1	Op0031f	1.25	0.66	1.06	0.52	**0.23**	*bioB*	Rmet_0117	4380	4453	Q1LS73 Biotin synthase
Rep1	Op0031f	1.29	0.75	1.09	0.80	**0.29**	*ycdW*	Rmet_0118	1842	2189	Q1LS72 D-isomer specific 2-hydroxyacid dehydrogenase, NAD-binding
Rep1	Op0031f	1.43	0.86	1.23	1.00	**0.41**	-	Rmet_0119	589	631	Q1LS71 YbaK/prolyl-tRNA synthetase associated region
Rep1	Op0031f	1.28	0.84	1.13	0.95	**0.46**	-	Rmet_0120	34	32	Q1LS70 Putative uncharacterized protein
Rep1	Op0072r	1.21	0.81	1.10	1.12	**0.43**	-	Rmet_0249	251	249	Q1LRU1 UBA/THIF-type NAD/FAD binding fold
Rep1	Op0075f	1.19	0.87	1.45	0.96	**0.46**	*coxA*	Rmet_0262	361	88	Q1LRS8 Cytochrome c oxidase, subunit I
Rep1	Op0119f	1.69	1.46	1.09	0.74	**0.35**	-	Rmet_0457	26	NF/NF	Q1LR83 Aminoglycoside phosphotransferase
Rep1	Op0149f	0.94	0.65	0.94	1.09	**0.43**	-	Rmet_0539	6581	7551	Q1LR01 Superoxide dismutase
Rep1	Op0259f	1.26	1.06	0.86	1.14	**0.48**	-	Rmet_0894	113	71	Q1LPZ6 Transcriptional regulator, LysR family
Rep1	Op0263f	1.41	1.96	1.48	1.14	**0.45**	*ilvC*	Rmet_0913	5505	6086	Q1LPX7 Ketol-acid reductoisomerase
Rep1	Op0267f	1.65	1.22	0.92	0.68	**0.39**	*nuoH*	Rmet_0934	538	200	Q1LPV6 NADH-quinone oxidoreductase subunit H
Rep1	Op0267f	1.33	1.03	0.95	0.91	**0.45**	*nuoI*	Rmet_0935	920	584	Q1LPV5 NADH-quinone oxidoreductase subunit I
Rep1	Op0267f	1.27	0.92	0.92	0.91	**0.50**	*nuoJ*	Rmet_0936	433	NF/NF	Q1LPV4 NADH-ubiquinone/plastoquinone oxidoreductase, chain 6
Rep1	Op0403f	1.38	1.75	0.88	0.89	**0.49**	*tsf*	Rmet_1436	4224	4521	Q1LNF7 Elongation factor Ts
Rep1	Op0525f	1.24	0.74	1.20	0.63	**0.50**	-	Rmet_1809	311	362	Q1LMD8 Thiamine pyrophosphate enzyme-like TPP-binding
Rep1	Op0570r	1.17	1.12	0.77	0.86	**0.37**	-	Rmet_1946	NeF	NeF	Q1LM01 Putative transmembrane protein
Rep1	Op0649f	1.20	0.94	0.89	0.91	**0.47**	*fumA*	Rmet_2273	3807	4273	Q1LL24 Fumarase
Rep1	Op0649f	1.25	0.77	0.88	1.23	**0.47**	*murI*	Rmet_2274	117	122	Q1LL23 Glutamate racemase
Rep1	Op0656r	1.03	0.89	0.87	0.80	**0.20**	-	Rmet_2299	NeF	NeF	Q8GQ11 Putative integral membrane protein / transporter
Rep1	Op0659f	1.22	0.97	1.10	0.74	**0.38**	*cadA*	Rmet_2303	82	NF/NF	A7HYL0 Heavy metal translocating P-type ATPase
Rep1	Op0843f	1.49	1.16	1.30	0.96	**0.49**	-	Rmet_2997	NeF	NeF	A9BTQ2 Putative uncharacterized protein
Rep1	Op0861f	1.10	1.56	1.07	0.99	**0.48**	*accB*	Rmet_3064	2052	2600	Q1LIU0 Biotin carboxyl carrier protein
Rep1	Op0969f	1.39	1.27	1.11	0.68	**0.40**	*gcvP*	Rmet_3482	372	407	Q1LHM2 Glycine dehydrogenase [decarboxylating]
Rep1	Op0979f	1.33	1.66	1.25	1.08	**0.49**	-	Rmet_3538	5550	7535	Q1LHG6 HU family DNA-binding protein
Rep1	Op0980r	1.32	1.57	1.22	1.00	**0.46**	-	Rmet_3539	NeF	NeF	Q1LHG5 Putative uncharacterized protein
Rep2	Op1008f	0.93	1.38	0.55	1.28	**0.18**	-	Rmet_3620	NF/NF	118	Q1LH84 Peptidase S1C, Do
Rep2	Op1008f	1.16	1.16	0.86	1.34	**0.43**	-	Rmet_3621	NeF	NeF	Q1LH83 Putative uncharacterized protein
Rep2	Op1110f	1.23	0.87	1.42	0.92	**0.48**	-	Rmet_3956	NeF	NeF	Q1LGA2 Cytochrome c oxidase, subunit I
Rep2	Op1322f	1.01	0.96	0.95	0.92	**0.25**	*czcI2*	Rmet_4595	NeF	NeF	Q1LEG9 Putative uncharacterized protein
Rep2	Op1322f	1.16	0.80	0.95	0.77	**0.02**	*czcC2*	Rmet_4596	147	72	Q1LEG8 Outer membrane efflux protein
Rep2	Op1322f	1.72	0.90	1.41	1.04	**0.10**	*czcB2'*	Rmet_4597	0	0	Q1LEG7 Secretion protein HlyD
Rep2	Op1323r	1.45	0.90	1.13	1.06	**0.13**	-	Rmet_4598	NF/NQ	NF/NF	B1LRU9 Tn3 family transposase
Rep2	Op1408f	1.70	1.18	1.66	0.85	0.47	*qseC*	Rmet_4830	111	NF/NF	Q1LDT4 Sensor protein
Rep2	Op1449r	1.29	1.58	1.06	0.68	**0.46**	-	Rmet_4936	NeF	NeF	Q1LDH8 Putative uncharacterized protein

Rep2	Op1478f	1.59	1.24	1.38	0.63	0.49	-	Rmet_5023	NeF	NeF	Q1LD91 AMP-dependent synthetase and ligase
Rep2	Op1544f	1.17	0.82	0.93	1.10	0.31	-	Rmet_5231	75	102	Q1LCN6 Transcriptional regulator, ArsR family
Rep2	Op1544f	1.26	0.62	0.98	0.78	0.30	*cydA*	Rmet_5232	53	28	Q1LCN5 Cytochrome bd ubiquinol oxidase, subunit I
Rep2	Op1544f	1.11	0.76	0.86	0.76	0.25	*cydB*	Rmet_5233	NeF	NeF	Q1LCN4 Cytochrome d ubiquinol oxidase, subunit II
Rep2	Op1544f	1.43	0.69	1.19	0.94	0.36	-	Rmet_5234	NeF	NeF	Q1LCN3 Putative uncharacterized protein
		**VF2.Up-regulated in Q(AE104:EDTA/Zn)**									
Rep1	Op0027f	1.24	1.40	0.53	0.93	2.16	-	Rmet_0094	485	539	Q1LS96 Putative uncharacterized protein
Rep1	Op0032r	1.41	1.02	1.01	0.66	2.11	-	Rmet_0123	NeF	NeF	Q1LS67 TonB-dependent receptor
Rep1	Op0071f	0.84	1.01	1.08	1.27	11.04	-	Rmet_0247	NeF	NeF	Q1LRU3 BFD-like (2Fe-2S)-binding region
Rep1	Op0088r	1.74	0.87	1.19	0.68	3.11	-	Rmet_0310	3865	3340	Q1LRN0 Peptidase C56, PfpI
Rep1	Op0109f	0.74	0.66	1.03	1.14	3.72	*gdhA*	Rmet_0398	343	628	Q1LRE2 Glu/Leu/Phe/Val dehydrogenase
Rep1	Op0116r	0.80	0.59	1.10	0.80	2.06	-	Rmet_0421	5059	2156	Q1LRB8 Putative uncharacterized protein
Rep1	Op0116r	0.82	0.68	1.01	0.91	2.01	-	Rmet_0422	4543	2044	Q1LRB7 Holliday junction ATP-dependent DNA helicase ruvB
Rep1	Op0159f	0.93	0.77	1.33	0.95	2.25	*fabI*	Rmet_0565	383	221	Q1LQX5 Enoyl-[acyl-carrier-protein] reductase (NADH)
Rep1	Op0159f	0.90	0.90	1.32	0.93	2.03	-	Rmet_0566	NeF	NeF	Q1LQX4 Putative uncharacterized protein
Rep1	Op0239f	1.35	1.26	1.21	0.94	38.18	-	Rmet_0837	20	NF/NF	Q1LQ53 TonB-dependent siderophore receptor
Rep1	Op0239f	1.35	0.96	1.19	0.79	39.11	-	Rmet_0838	58	67	Q1LQ52 PKHD-type hydroxylase Rmet_0838
Rep1	Op0239f	1.32	1.16	1.18	1.10	31.14	-	Rmet_0839	NeF	NeF	Q1LQ51 FMN-dependent alpha-hydroxy acid dehydrogenase
Rep1	Op0251f	0.74	1.17	1.03	0.86	2.28	-	Rmet_0869	257	185	Q1LQ21 FAD dependent oxidoreductase
Rep1	Op0295f	1.19	1.17	0.72	1.05	2.64	-	Rmet_1024	204	401	Q1LPL6 Transcriptional regulator, BadM/Rrf2 family
Rep1	Op0315f	0.77	1.31	1.17	1.19	2.74	-	Rmet_1092	NeF	NeF	Q1LFA7 Anion transporter
Rep1	Op0315f	0.69	1.19	1.30	0.97	4.88	*adh*	Rmet_1093	196	386	Q1LPE7 Alcohol dehydrogenase GroES-like protein
Rep1	Op0319f	1.19	1.39	1.36	0.89	11.40	-	Rmet_1105	NeF	NeF	Q1LPD5 Putative uncharacterized protein
Rep1	Op0319f	1.16	1.34	1.11	0.89	4.58	-	Rmet_1106	NeF	NeF	Q1LPD4 PepSY-associated TM helix
Rep1	Op0319f	1.15	1.32	1.15	0.95	4.45	-	Rmet_1107	NeF	NeF	Q1LPD3 Putative uncharacterized protein
Rep1	Op0319f	1.12	1.14	1.13	0.80	8.77	-	Rmet_1108	NeF	NeF	Q1LPD2 TonB-dependent siderophore receptor
Rep1	Op0320r	0.88	1.13	0.99	0.93	6.25	-	Rmet_1109	NF/NF	NF/NF	Q1LPD1 Putative uncharacterized protein
Rep1	Op0320r	0.74	1.20	0.95	0.90	5.29	*lysA*	Rmet_1110	NF/NF	NF/NF	Q1LPD0 Orn/DAP/Arg decarboxylase 2
Rep1	Op0320r	0.93	1.33	1.14	0.96	9.21	*hpcH*	Rmet_1111	NF/NF	45	Q1LPC9 HpcH/HpaI aldolase
Rep1	Op0320r	0.75	1.33	1.03	0.93	5.10	-	Rmet_1112	22	NF/NF	Q1LPC8 IucA/IucC
Rep1	Op0320r	0.77	1.22	1.00	0.93	9.11	-	Rmet_1113	221	NF/NF	Q1LPC7 IucA/IucC
Rep1	Op0320r	0.78	1.03	0.94	0.67	7.45	-	Rmet_1114	NeF	NeF	Q1LPC6 Major facilitator superfamily MFS_1
Rep1	Op0320r	0.83	1.14	1.04	0.94	9.34	-	Rmet_1115	NeF	NeF	Q1LPC5 IucA/IucC
Rep1	Op0320r	0.83	1.28	1.07	0.97	6.56	*ocd*	Rmet_1116	299	373	Q1LPC4 Ornithine cyclodeaminase
Rep1	Op0320r	0.81	1.12	1.07	0.77	13.15	*cysK*	Rmet_1117	NF/NF	NF/NF	Q44004 Cysteine synthase
Rep1	Op0320r	0.90	1.11	1.01	1.17	8.27	-	Rmet_1118	209	84	Q1LPC2 TonB-dependent siderophore receptor
Rep1	Op0320r	1.12	0.82	1.11	0.78	3.15	*rsiA*	Rmet_1119	1538	NF/NF	Q1LPC1 Putative FecR
Rep1	Op0320r	0.95	0.91	0.85	0.94	5.17	*rpoI*	Rmet_1120	NeF	NeF	Q1LPC0 Sigma-24 (FecI-like)
Rep1	Op0333f	1.51	0.93	0.67	0.74	2.13	*thrS*	Rmet_1160	1119	1126	Q1LP80 Threonyl-tRNA synthetase / Ser-tRNA(Thr) hydrolase
Rep1	Op0384r	1.20	1.15	1.18	0.89	3.49	-	Rmet_1365	NeF	NeF	Q1LNM8 PepSY-associated TM helix
Rep1	Op0384r	1.23	1.18	1.05	0.64	2.57	-	Rmet_1366	NeF	NeF	Q1LNM7 Putative transmembrane protein
Rep1	Op0384r	1.41	1.10	1.18	0.68	2.06	-	Rmet_1367	NeF	NeF	Q1LNM6 Putative lipoprotein transmembrane
Rep1	Op0481f	0.60	1.11	1.13	0.89	6.11	-	Rmet_1695	197	291	Q1LMQ0 Putative uncharacterized protein
Rep1	Op0481f	0.61	1.12	1.19	0.88	3.40	-	Rmet_1696	109	394	Q1LMP9 Putative uncharacterized protein
Rep1	Op0481f	0.70	1.23	0.96	1.03	3.35	-	Rmet_1697	NeF	NeF	Q1LMP8 Sensor protein
Rep1	Op0481f	0.75	1.27	1.15	1.05	3.50	-	Rmet_1698	NeF	NeF	Q1LMP7 Two component transcriptional regulator, LuxR family
Rep1	Op0481f	0.72	1.07	1.19	0.97	7.42	-	Rmet_1699	NeF	NeF	Q1LMP6 Response regulator receiver domain protein (CheY-like)
Rep1	Op0481f	0.61	1.00	1.03	0.86	7.42	-	Rmet_1700	343	167	Q1LMP5 Putative uncharacterized protein
Rep1	Op0481f	0.86	1.60	1.48	1.14	4.88	-	Rmet_1701	434	166	Q1LMP4 Secretion protein HlyD
Rep1	Op0481f	0.60	1.27	0.99	0.94	10.33	-	Rmet_1702	226	37	Q1LMP3 Hydrophobe/amphiphile efflux-1 HAE1
Rep1	Op0481f	0.82	1.26	1.12	1.03	5.89	-	Rmet_1703	NeF	NeF	Q1LMP2 RND efflux system, outer membrane lipoprotein, NodT

Rep1	Op0481f	0.78	1.87	1.39	1.14	**7.15**	-	Rmet_1704	392	156	Q1LMP1	Putative uncharacterized protein
Rep1	Op0481f	0.82	1.43	1.36	1.23	**8.04**	-	Rmet_1706	408	312	Q1LMN9	Putative uncharacterized protein
Rep1	Op0482r	0.78	1.22	1.25	0.99	**4.86**	-	Rmet_1707	NeF	NeF	Q1LMN8	Putative uncharacterized protein
Rep1	Op0483f	0.77	1.26	1.13	1.02	**2.36**	-	Rmet_1709	NeF	NeF	Q1LMN6	Putative uncharacterized protein
Rep1	Op0507f	0.57	1.10	1.40	0.95	**8.66**	*gadB*	Rmet_1766	1046	898	Q1LMI1	Glutamate decarboxylase
Rep1	Op0519f	1.13	0.95	1.41	1.12	**2.09**	-	Rmet_1791	65	40	Q1LMF6	Secretion protein HlyD
Rep1	Op0527f	1.60	1.13	1.58	0.81	**27.51**	-	Rmet_1819	NeF	NeF	Q1LMC8	TonB-dependent siderophore receptor
Rep1	Op0527f	1.70	0.95	1.41	1.00	**3.25**	*purT*	Rmet_1820	1022	1078	Q1LMC7	Phosphoribosylglycinamide formyltransferase 2
Rep1	Op0555f	0.86	1.29	1.36	0.82	**4.49**	-	Rmet_1904	NF/NF	163	Q1LM43	Putative uncharacterized protein
Rep1	Op0579f	0.70	1.29	1.41	0.59	**8.59**	*asdA*	Rmet_1984	3127	2969	Q1LLW3	Aminotransferase
Rep1	Op0579f	0.63	1.11	1.24	0.89	**6.77**	-	Rmet_1985	281	710	Q1LLW2	YidE/YbjL duplication
Rep1	Op0580r	0.55	0.87	0.98	0.95	**4.13**	-	Rmet_1986	NeF	NeF	Q1LLW1	Putative uncharacterized protein
Rep1	Op0651f	0.92	0.76	0.95	0.86	**13.58**	-	Rmet_2276	NeF	NeF	Q1LL21	Putative uncharacterized protein
Rep1	Op0651f	0.82	0.70	0.84	0.81	**14.41**	-	Rmet_2277	44	NF/NF	Q1LL20	TonB-like protein
Rep1	Op0651f	0.90	0.90	0.91	0.89	**15.93**	-	Rmet_2278	445	173	Q1LL19	MotA/TolQ/ExbB proton channel
Rep1	Op0651f	1.00	1.01	0.99	1.14	**15.38**	-	Rmet_2279	188	109	Q1LL18	Biopolymer transport protein ExbD/TolR
Rep1	Op0651f	0.97	1.02	1.26	0.80	**40.88**	-	Rmet_2280	NF/NF	48	Q1LL17	Putative uncharacterized protein
Rep1	Op0652r	0.96	0.65	0.88	0.76	**2.82**	-	Rmet_2282	NeF	NeF	Q1LL15	Virulence protein
Rep2	Op1019r	1.22	0.98	1.07	0.72	**2.43**	-	Rmet_3649	NeF	NeF	Q1LH55	Putative uncharacterized protein
Rep2	Op1026f	1.89	1.63	1.37	1.48	**2.05**	*cheA*	Rmet_3689	81	NF/NF	Q1LH15	CheA signal transduction histidine kinases
Rep2	Op1102f	0.86	0.99	1.06	1.04	**3.67**	-	Rmet_3941	NeF	NeF	Q1LGB7	Putative uncharacterized protein
Rep2	Op1123r	0.86	1.08	0.91	0.84	**2.35**	*rskA*	Rmet_4000	31	NF/NF	Q1LG58	FecR family protein
Rep2	Op1123r	1.18	1.08	1.41	0.82	**3.10**	*rpoK*	Rmet_4001	NeF	NeF	Q1LG57	Sigma-24 (FecI-like)
Rep2	Op1251r	0.55	1.07	0.93	0.94	**2.07**	-	Rmet_4400	2088	287	Q1LF11	Putative uncharacterized protein
Rep2	Op1281r	0.95	1.33	0.96	0.88	**2.13**	*rsjA*	Rmet_4498	85	101	Q1LER3	Putative FecR
Rep2	Op1331r	1.58	1.25	0.62	0.61	**2.02**	-	Rmet_4619	NeF	NeF	Q1LEE5	RND efflux system, outer membrane lipoprotein, NodT
Rep2	Op1357r	0.75	0.81	1.07	1.00	**2.03**	-	Rmet_4692	NeF	NeF	Q1LE72	Putative uncharacterized protein
Rep2	Op1412f	0.67	1.13	1.16	0.96	**3.92**	-	Rmet_4847	58	NF/NF	Q1LDR7	YidE/YbjL duplication
Rep2	Op1600f	1.29	1.33	1.11	0.96	**37.04**	-	Rmet_5373	72	NF/NF	Q1LC94	TonB-dependent receptor
Rep2	Op1600f	1.16	1.18	1.21	0.75	**444.51**	-	Rmet_5374	NeF	NeF	Q1LC93	Putative uncharacterized protein
Rep2	Op1600f	1.44	1.13	1.11	0.76	**144.27**	-	Rmet_5375	NeF	NeF	Q1LC92	Haemin-degrading
Rep2	Op1600f	1.54	1.22	1.08	0.87	**92.78**	*hmuT*	Rmet_5376	NeF	NeF	Q1LC91	Periplasmic binding protein
Rep2	Op1600f	1.53	1.23	1.23	0.81	**280.57**	*hmuU*	Rmet_5377	NeF	NeF	Q1LC90	Transport system permease protein
Rep2	Op1600f	1.71	1.29	1.26	0.94	**25.44**	*hmuV*	Rmet_5378	NeF	NeF	Q1LC89	Hemin import ATP-binding protein hmuV
Rep2	Op1601r	1.59	1.30	1.12	0.85	**21.11**	-	Rmet_5379	NeF	NeF	Q1LC88	Short-chain dehydrogenase/reductase SDR
Rep2	Op1601r	1.54	1.33	1.10	0.94	**3.70**	-	Rmet_5380	NeF	NeF	Q1LC87	Putative uncharacterized protein
Rep2	Op1608f	0.59	1.09	0.96	0.78	**2.14**	-	Rmet_5402	247	131	Q1LC65	Beta-lactamase-like protein
Rep2	Op1610f	0.62	1.14	1.19	0.77	**2.08**	-	Rmet_5405	26	NF/NF	Q1LC62	Putative periplasmic protein
Rep2	Op1610f	0.70	1.17	1.19	1.04	**3.24**	-	Rmet_5406	110	NF/NF	Q1LC61	Putative uncharacterized protein
Rep2	Op1610f	0.65	1.17	1.02	0.82	**2.78**	-	Rmet_5408	NeF	NeF	Q1LC59	Outer membrane efflux protein
Rep2	Op1610f	0.62	1.16	0.98	0.85	**2.62**	-	Rmet_5409	63	243	Q1LC58	Putative uncharacterized protein
Rep2	Op1612f	0.61	1.11	1.01	0.78	**5.09**	*aslA*	Rmet_5416	543	351	Q1LC51	Sulfatase
Rep2	Op1628f	0.74	0.71	0.80	1.01	**2.41**	-	Rmet_5484	441	670	Q1LBY3	Putative uncharacterized protein
Rep2	Op1660f	0.65	1.04	1.04	1.14	**13.87**	-	Rmet_5578	1738	759	Q1LBN9	Putative uncharacterized protein
Rep2	Op1660f	0.77	1.25	1.13	1.17	**11.76**	-	Rmet_5579	NF/NF	NF/NF	Q1LBN8	Cytochrome B561
Rep2	Op1668f	0.74	0.94	1.12	0.91	**2.29**	-	Rmet_5600	94	113	Q1LBL7	Ankyrin
Rep2	Op1681r	1.05	1.02	1.30	0.74	**2.35**	-	Rmet_5636	NeF	NeF	Q1LBI1	Flavodoxin/nitric oxide synthase
Rep2	Op1681r	0.61	1.11	1.13	0.93	**2.28**	-	Rmet_5637	NeF	NeF	Q1LBI0	ApbE-like lipoprotein
Rep2	Op1681r	0.77	1.03	1.14	0.95	**3.14**	-	Rmet_5638	285	293	Q1LBH9	Putative uncharacterized protein
Rep2	Op1681r	0.76	1.04	1.05	0.91	**2.27**	-	Rmet_5639	139	84	Q1LBH8	Putative uncharacterized protein
Rep2	Op1718f	0.95	1.19	1.63	1.10	**57.71**	*furB*	Rmet_5746	23	50	Q1LB71	Ferric uptake regulator, Fur family

Rep2	Op1718f	1.03	1.05	1.80	0.99	**13.23**	-	Rmet_5747	NeF	NeF	Q1LB70 Putative membrane protein
Rep2	Op1719r	0.86	0.94	1.25	0.94	**2.87**	-	Rmet_5748	NeF	NeF	Q1LB69 Putative uncharacterized protein
Rep2	Op1738f	0.51	0.74	0.83	0.63	**2.85**	-	Rmet_5780	NeF	NeF	Q1LB37 Putative uncharacterized protein
Rep2	Op1738f	0.61	1.08	1.05	0.87	**2.14**	-	Rmet_5781	NeF	NeF	Q1LB36 Putative uncharacterized protein
Rep2	Op1785r	0.88	0.82	0.96	0.85	**2.07**	-	Rmet_5890	100	37	Q1LAS7 Ferrous iron transport protein B
Rep2	Op1785r	1.03	0.81	1.12	0.83	**2.08**	-	Rmet_5891	35	164	Q1LAS6 FeoA

VF3. Notregulated in Q(AE104:EDTA/Zn)

5255 genes
