# Supplementary material for: Characterization of the Δ7 Mutant of Cupriavidus metallidurans with Deletions of Seven Secondary Metal Uptake Systems
Source: mSystems. 2016 Feb 25;1(1):e00004-16. doi: 10.1128/mSystems.00004-16 (PMC5069749; doi:10.1128/mSystems.00004-16)
Supplement: Table S2 [file sys001162005st2.pdf]

Supplementary Table S2. Regulation of *lacZ* fusions with genes that might be involved in zinc homoeostasis[a].

Gene	Condition	Parent, -fold change		
		AE104	Δ*zupT*	Δ7
Rmet_0837	0	1.00	1.26	**0.42**
4.83±0.11	10 µM Cd(II)	0.82	1.05	**0.34**
	125 µM Cd(II)	1.10	1.21	0.54
	10 µM Co(II)	1.00	1.08	**0.27**
	125 µM Co(II)	0.87	1.21	**0.35**
	50 µM EDTA	1.47	**3.14**	1.12
	1 mM EDTA	**51.03**	**50.85**	**39.69**
	10 µM Zn(II)	0.97	0.93	**0.29**
	125 µM Zn(II)	**5.61**	**4.87**	**2.18**
Rmet_1098	0	1.00	**3.70**	n.d.
1.20±0.16	10 µM Cd(II)	1.43	**7.21**	n.d.
	125 µM Cd(II)	1.04	**4.02**	n.d.
	10 µM Co(II)	0.89	**2.96**	n.d.
	125 µM Co(II)	0.82	*2.83*	n.d.
	50 µM EDTA	**20.34**	**96.02**	n.d.
	1 mM EDTA	**66.09**	**69.67**	n.d.
	10 µM Zn(II)	0.82	**2.61**	n.d.
	125 µM Zn(II)	0.71	**3.31**	n.d.
Rmet_1106	0	1.00	0.65	n.d.
6.41±0.08	10 µM Cd(II)	0.87	0.56	n.d.
	125 µM Cd(II)	0.66	**0.17**	n.d.
	10 µM Co(II)	1.12	0.61	n.d.
	125 µM Co(II)	0.93	**0.27**	n.d.
	50 µM EDTA	1.15	0.59	n.d.
	1 mM EDTA	**4.07**	1.96	n.d.
	10 µM Zn(II)	1.06	0.64	n.d.
	125 µM Zn(II)	0.90	**0.23**	n.d.
Rmet_1114	0	1.00	0.71	**0.50**
26.2±2.5	10 µM Cd(II)	0.82	0.78	0.58
	125 µM Cd(II)	0.92	0.82	0.54
	10 µM Co(II)	0.94	0.71	**0.49**
	125 µM Co(II)	0.94	0.87	**0.47**
	50 µM EDTA	**3.17**	**4.49**	1.21
	1 mM EDTA	**7.11**	**6.36**	**4.43**
	10 µM Zn(II)	0.80	0.64	**0.48**
	125 µM Zn(II)	1.07	1.01	0.52

Rmet_1794	0	1.00	0.82	**0.44**
15.0±0.3	10 µM Cd(II)	0.85	0.84	**0.37**
	125 µM Cd(II)	0.54	0.68	**0.25**
	10 µM Co(II)	0.78	0.80	**0.34**
	125 µM Co(II)	0.64	0.66	**0.29**
	50 µM EDTA	0.90	0.95	**0.43**
	1 mM EDTA	0.95	1.01	**0.30**
	10 µM Zn(II)	1.05	0.90	**0.40**
	125 µM Zn(II)	0.66	0.75	**0.30**
Rmet_1819	0	1.00	**3.45**	n.d.
1.49±0.04	10 µM Cd(II)	0.81	*2.87*	n.d.
	125 µM Cd(II)	1.26	*2.59*	n.d.
	10 µM Co(II)	1.02	**4.00**	n.d.
	125 µM Co(II)	1.10	*3.00*	n.d.
	50 µM EDTA	1.13	**3.71**	n.d.
	1 mM EDTA	**79.18**	**54.51**	n.d.
	10 µM Zn(II)	0.76	**3.28**	n.d.
	125 µM Zn(II)	**3.28**	*4.67*	n.d.
Rmet_3361	0	1.00	0.83	0.81
12.7±2.1	10 µM Cd(II)	0.81	0.71	0.78
	125 µM Cd(II)	**0.43**	**0.43**	0.89
	10 µM Co(II)	0.86	0.74	0.73
	125 µM Co(II)	0.64	0.64	0.63
	50 µM EDTA	0.97	0.88	0.83
	1 mM EDTA	0.91	0.91	0.79
	10 µM Zn(II)	0.69	0.73	0.83
	125 µM Zn(II)	**0.47**	**0.47**	0.65
Rmet_5377	0	1.00	**5.68**	*0.66*
0.78±0.22	10 µM Cd(II)	1.25	**5.18**	n.d.
	125 µM Cd(II)	0.60	0.84	**0.41**
	10 µM Co(II)	0.80	**4.89**	n.d.
	125 µM Co(II)	0.79	1.51	**0.50**
	50 µM EDTA	**5.30**	**5.76**	n.d.
	1 mM EDTA	**107.83**	**81.89**	**82.77**
	10 µM Zn(II)	1.14	**3.50**	n.d.
	125 µM Zn(II)	*2.19*	*2.12*	**0.28**

Rmet_5404	0	1.00	0.64	0.83
16.3±2.1	10 µM Cd(II)	0.76	0.50	0.61
	125 µM Cd(II)	**0.47**	**0.49**	**0.49**
	10 µM Co(II)	0.89	0.59	0.79
	125 µM Co(II)	0.77	0.58	0.72
	50 µM EDTA	0.80	0.68	0.73
	1 mM EDTA	0.82	0.77	0.66
	10 µM Zn(II)	0.71	0.55	0.70
	125 µM Zn(II)	0.57	**0.48**	0.59
Rmet_5640	0	1.00	0.75	0.55
10.5±0.8	10 µM Cd(II)	0.84	0.70	**0.46**
	125 µM Cd(II)	0.61	0.81	0.61
	10 µM Co(II)	0.90	0.62	0.55
	125 µM Co(II)	0.70	1.00	0.53
	50 µM EDTA	1.02	0.93	0.63
	1 mM EDTA	**5.71**	**5.63**	**4.15**
	10 µM Zn(II)	0.84	0.63	0.51
	125 µM Zn(II)	1.46	1.67	0.66
Rmet_5747	0	1.00	0.64	n.d.
6.15±0.35	10 µM Cd(II)	0.98	0.59	n.d.
	125 µM Cd(II)	0.82	0.69	n.d.
	10 µM Co(II)	1.06	0.72	n.d.
	125 µM Co(II)	0.94	1.06	n.d.
	50 µM EDTA	1.06	1.07	n.d.
	1 mM EDTA	**9.67**	**6.63**	n.d.
	10 µM Zn(II)	1.14	0.51	n.d.
	125 µM Zn(II)	1.85	1.21	n.d.

[a]The values below the gene names are the specific activities in AE104 cells cultivated in TMM without further additions. All other values were divided by this basic activity. Red and bold-faced numbers indicate significant (D >1) down-regulations, green and bold-faced numbers significant up-regulations
