# Supplementary material for: Characterization of the Δ7 Mutant of Cupriavidus metallidurans with Deletions of Seven Secondary Metal Uptake Systems
Source: mSystems. 2016 Feb 25;1(1):e00004-16. doi: 10.1128/mSystems.00004-16 (PMC5069749; doi:10.1128/mSystems.00004-16)
Supplement: Table S3 [file sys001162005st3.pdf]

Supplementary Table S3. Primers and plasmids used.

Name	5´→3´-sequence	comment	work
MgtA lacZ1 SphI	CAT GCA TGC-GCG GAT GAA ATG GCG CGA ACA GC	LacZ primer *mgtA*	this work
MgtA lacZ 2 XbaI	GCC TCT AGA-ATT GCGACATGCGTTCAGGGCTT	LacZ primer *mgtA*	this work
MgtB lacZ1 SphI	GAA GCA TGC-AGT CTG TTC CTG TGG GAA CTA TC	LacZ primer *mgtB*	this work
MgtB lacZ2 XbaI	TCG TCT AGA-GGC AGA TTC AAT CGG CAG CCAG AC	LacZ primer *mgtB*	this work
KO Rmet_5396_1 MunI	AAA CAA TTG GAG TAC ATG CAG GCC TAC GA	*cre-lox* primer *mgtA*	this work
KO Rmet_5396_4 AgeI	AAA ACC GGT AGG CTG TAC GGC GAA TCG GT	*cre-lox* primer *mgtA*	this work
KO Rmet_5396_3 ApaI	AAA GGG CCC CCT GAA CGC ATG TCG CAA TC	*cre-lox* primer *mgtA*	this work
KO Rmet_5396_2 NotI	AAA GCG GCC GCC GGT ATC CGC TTC ACT CAT C	*cre-lox* primer *mgtA*	this work
KO Rmet_2211_1 AgeI	AAA ACC GGT ATG CGC CGT TGA CGG TGA CT	*cre-lox* primer *mgtB*	this work
KO Rmet_2211_4 MunI	AAA CAA TTG TGC CAT GTT GCG GAT CGT CT	*cre-lox* primer *mgtB*	this work
KO Rmet_2211_2 ApaI	AAA GGG CCC TCG GTG AGC GTG TGC CAG AT	*cre-lox* primer *mgtB*	this work
KO Rmet_2211 3 NotI	AAA GCG GCC GCT GGC TGC CGA TTG AAT CTG C	*cre-lox* primer *mgtB*	this work
Rmet_5377 PstI Dis	AAA CTG CAG CAG GCG CTC TTC CGC AAT C	LacZ/Dis primer Rmet_5377	this work
Rmet_5377 XbaI Dis	AAA TCT AGA CAC CAG CGG CAC AAG CCA C	LacZ/Dis primer Rmet_5377	this work
Rmet_1819 PstI Dis	AAA CTG CAG ACG GGC CTG CGT TAC AAC C	LacZ/Dis primer Rmet_1819	this work
Rmet_1819 XbaI Dis	AAA TCT AGA GTC GCT GGC ACG GTA CTT G	LacZ/Dis primer Rmet_1819	this work
Rmet_1106 PstI Dis	AAA CTG CAG CAT CGG CGC ATC TTC ACG G	LacZ/Dis primer Rmet_1106	this work
Rmet_1106 XbaI Dis	AAA TCT AGA AGG GAG ACG GTG GCA TTG G	LacZ/Dis primer Rmet_1106	this work
Rmet_1114 PstI Dis	AAA CTG CAG GGC CAC TTC GCA GCA TGT C	LacZ/Dis primer Rmet_1104	this work
Rmet_1114 XbaI Dis	AAA TCT AGA CCA GCG GCG CAG CAT CAG	LacZ/Dis primer Rmet_1104	this work
Rmet_0837 PstI Dis	AAA CTG CAG CAA GCG GGC GAA CCT GAA C	LacZ/Dis/RT-PCR primer Rmet_0837	this work
Rmet_0837 XbaI Dis	AAA TCT AGA ATG GGC GTC GGT GAT GTC G	LacZ/Dis/Rt-PCR primer Rmet_0837	this work
Rmet_5404 PstI Dis	AAA CTG CAG GCC GCC GAC GAT CTA TTT C	LacZ/Dis primer Rmet_5404	this work
Rmet_5404 XbaI Dis	AAA TCT AGA CAG AAC CGC GGC CAG TTG	LacZ/Dis primer Rmet_5404	this work
Rmet_1098 PstI Dis	AAA CTG CAG GGC CGC AGT CTC AAT GAG G	LacZ/Dis/RT-PCR primer Rmet_1098	(1)
Rmet_1098 XbaI Dis	AAA TCT AGA GGG CGC TTT CGA TGC TTC C	LacZ/Dis/RT-PCR primer Rmet_1098	(1)
Rmet_5747 PstI Dis	AAA CTG CAG GCC TCC GCC TCC GCA ATG	LacZ/Dis primer Rmet_5747	this work
Rmet_5747 XbaI Dis	AAA TCT AGA CGT GCC AGC CGA CGA CAA C	LacZ/Dis primer Rmet_5747	this work
Rmet_5640 PstI Dis	AAA CTG CAG CGC GAT CTG CCT GAT CGG	LacZ/Dis/RT-PCR primer Rmet_5640	this work
Rmet_5640 XbaI Dis	AAA TCT AGA CTG GAC CTC GCC GTC TTC	LacZ/Dis/RT-PCR primer Rmet_5640	this work
Rmet_1794 PstI Dis	AAA CTG CAG GTC GTG CTG ATC CGC AAG G	LacZ/Dis/RT-PCR primer Rmet_1794	this work
Rmet_1794 XbaI Dis	AAA TCT AGA CAG TCC CGC TGG GCA AAT C	LacZ/Dis/RT-PCR primer Rmet_1794	this work
Rmet_3361 PstI Dis	AAA CTG CAG CTG ACG CCA ATC GCA GTC G	LacZ/Dis primer Rmet_3361	this work
Rmet_3361 XbaI Dis	AAA TCT AGA GCG CTG ACT CCG AAT ACC G	LacZ/Dis primer Rmet_3361	this work
rpoZRalme 28 up qPCR	CTG AAA CAC ATC CCG AAC C	RT primer *rpoZ*	(2)
rpoZRalme 120 down qPCR	ACG GTG GGC TTG TCC TTT G	RT primer *rpoZ*	(2)
Rmet_5890 PstI Dis	AAACTGCAGCACCGCGCAGTTCGAGGAC	RT primer Rmet_5890, *feoB*	this work
Rmet_5890 XbaI Dis	AAATCTAGAGGCCCGCGCTCATCATCAG	RT primer Rmet_5890, *feoB*	this work
qPCR hoxN2. up 1556	TGG CAT CAA CGG GCT GTG GA	RT primer Rmet_1533, *hoxN*	this work
qPCR hoxN2. down 1739	TGA CGC ACA CCA CGG ACA GC	RT primer Rmet_1533, *hoxN*	this work

Supplementary Table S3 cont.

Name	Relevant markers	Description	TCDB (3)	Reference
Plasmids				
pECD794-1	*lacZ*, derivate of pLO2			(4)
pCM157	Contains Cre recombinase			(5)
pECD1003	Mutant *lox* sites, derivate of pECD889			(4)
pVDZ′2	Broad host range expression vector			(6)
pDNA130	pVDZ′2::*czcCBAD´*			(7)
pECD1258	pECD1003 construct for *mgtB* deletion by *cre-lox* system			This study
pECD1259	pECD794 construct for *mgtA* disruption			This study
pECD1492	pECD794 construct for *mgtB* disruption			This study
pECD1488	pECD794 construct for *mgtA* LacZ Reportergen			This study
pECD1489	pECD794 construct for *mgtB* LacZ Reportergen			This study
pECD1205	pECD794 construct for Rmet_0837 LacZ/Disruption Reportergen			This study
pECD1204	pECD794 construct for Rmet_1098 LacZ/Disruption Reportergen			This study
pECD1210	pECD794 construct for Rmet_1106 LacZ/Disruption Reportergen			This study
pECD1208	pECD794 construct for Rmet_1114 LacZ/Disruption Reportergen			This study
pECD1207	pECD794 construct for Rmet_1794 LacZ/Disruption Reportergen			This study
pECD1203	pECD794 construct for Rmet_1819 LacZ/Disruption Reportergen			This study
pECD1213	pECD794 construct for Rmet_3361 LacZ/Disruption Reportergen			This study
pECD1202	pECD794 construct for Rmet_5377 LacZ/Disruption Reportergen			This study
pECD1211	pECD794 construct for Rmet_5404 LacZ/Disruption Reportergen			This study
pECD1206	pECD794 construct for Rmet_5640 LacZ/Disruption Reportergen			This study
pECD1209	pECD794 construct for Rmet_5747 LacZ/Disruption Reportergen			This study

References to the Supplementary Material

1. **Herzberg M, Schüttau M, Reimers M, Grosse C, Schlegel HG, and Nies DH.** 2015. Synthesis of nickel-iron hydrogenase in *Cupriavidus metallidurans* is controlled by metal-dependent silencing and un-silencing of genomic islands. Metallomics **7:**632 - 649.
2. **Große C, Friedrich S, and Nies DH.** 2007. Contribution of extracytoplasmic function sigma factors to transition metal homeostasis in *Cupriavidus metallidurans* strain CH34. J Mol Microbiol Biotechnol **12:**227-240.
3. **Saier MHJ, Tran CV, and Barabote RD.** 2006. TCDB: the Transporter Classification Database for membrane transport protein analyses and information. Nucl Acid Res **34:**D181-D186.
4. **Scherer J, and Nies DH.** 2009. CzcP is a novel efflux system contributing to transition metal resistance in *Cupriavidus metallidurans* CH34. Mol Microbiol **73:**601-621.
5. **Marx CJ, and Lidstrom ME.** 2002. Broad-host-range *cre-lox* system for antibiotic marker recycling in gram-negative bacteria. Biotechniques. **33:**1062-1067.
6. **Deretic V, Chandrasekharappa S, Gill JF, Chatterjee DK, and Chakrabarty A.** 1987. A set of cassettes and improved vectors for genetic and biochemical characterization of *Pseudomonas* genes. Gene **57:**61-72.
7. **Nies DH, Nies A, Chu L, and Silver S.** 1989. Expression and nucleotide sequence of a plasmid-determined divalent cation efflux system from *Alcaligenes eutrophus.* Proc Natl Acad Sci U S A **86:**7351-7355.
